# Neurodevelopmental impact of mining-related contamination in children from the Sonora river basin

**DOI:** 10.3389/fped.2025.1681071

**Published:** 2025-12-05

**Authors:** Diana Mejía-Cruz, Laurent Ávila-Chauvet, Agustín Robles-Morua, Joselinn Murataya-Gutierrez

**Affiliations:** 1 Psychology Department, Sonora Institute of Technology, Obregón City, Mexico; 2 Water and Environmental Sciences, Sonora Institute of Technology, Obregón City, Mexico

**Keywords:** neurodevelopment, environmental contamination, mining exposure, children, executive functioning

## Abstract

**Introduction:**

In August 2014, the Buenavista del Cobre mine in northern Mexico released over 40,000 m^3^ of acidified copper sulfate into the Sonora River, causing long-term environmental contamination. Children in nearby communities face increased neurodevelopmental risks due to prolonged exposure to toxic metals such as lead and arsenic. This study examines cognitive and executive functioning outcomes in exposed children, considering the roles of nutrition, parenting practices, and prenatal biological risk factors.

**Methods:**

A cross-sectional study was conducted with 238 children aged 5–13 years: 215 from mining-impacted communities and 23 from the non-exposed control community of Álamos. Children completed selected BANETA neuropsychological subtests (e.g., attention, academic skills, motor coordination), while caregivers completed the BRIEF-P, Parenting Practices Inventory (IPC), Mini Nutritional Assessment (MNA-SF), and a biological risk screening based on the Child Development Assessment (EDI). Standardized *z*-scores and non-parametric tests were used for group comparisons and correlational analyses.

**Results:**

Children from exposed communities showed significantly greater executive functioning difficulties, particularly in behavioral, emotional, and cognitive regulation. BANETA scores revealed lower attention, more omission errors, and poorer writing and motor performance. Executive deficits were significantly associated with lower nutritional status and greater biological risk. Punitive parenting practices correlated with poorer regulation, while supportive parenting was linked to better academic performance.

**Discussion:**

These findings support a multifactorial model where environmental, biological, and psychosocial stressors interact to adversely affect child development. Results underscore the need for integrated public health strategies, combining environmental remediation, educational support, nutrition, and caregiver-focused interventions in vulnerable communities.

## Introduction

**Figure 1:**
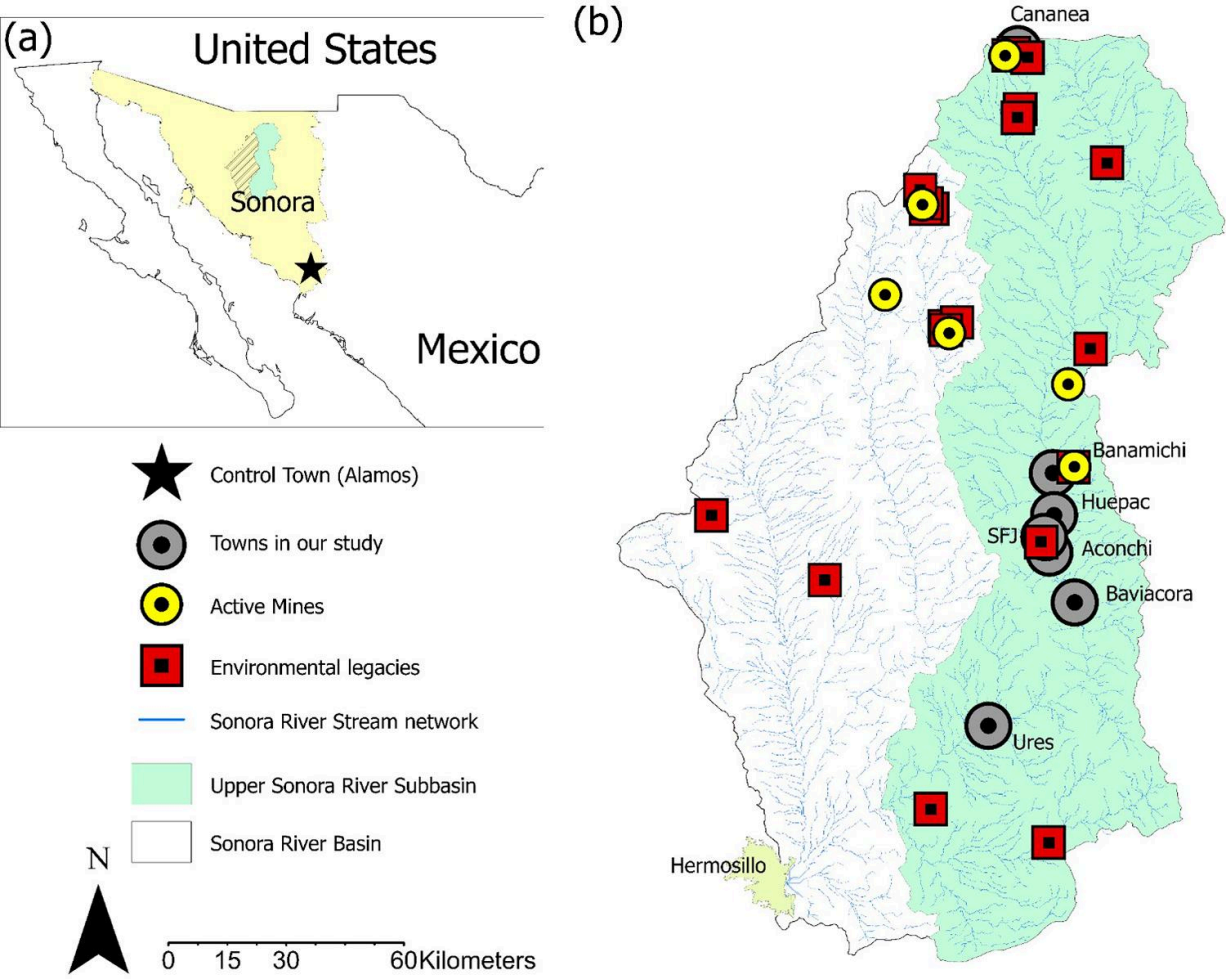
Geographic context and distribution of environmental risks in Sonora river basin. **(a)** The map shows the location of the state of Sonora in northwestern Mexico. **(b)** Detailed map of the Upper Sonora River Basin indicating the location of towns included in the study (gray circles), active mines (yellow circles with black outlines), and sites with environmental legacies (red squares with black outlines). The river network is shown in blue, while the shaded green area represents the subbasin under study.

On August 6, 2014, Mexico experienced one of the worst environmental disasters in its recent history. A spill of 40,000 m^3^ of sulfuric acid and copper sulfate leachate from the Buenavista del Cobre mine was released into the Sonora River Basin (SRB), located in northwestern Mexico (Figure 1). The spill severely affected public health, degraded biodiversity, and disrupted productive activities throughout the area (1). Concentrations of heavy metals such as arsenic (As), lead (Pb), copper (Cu), zinc (Zn), and other elements in groundwater and surface water have exceeded the safety thresholds established by the Official Mexican Standard for human water consumption (2). Additionally, elevated levels of these same elements have been reported in the soil and food sources of surrounding communities (3–5). Evidence of human exposure is further supported by biomonitoring data from 1,615 individuals living along the Sonora River, 80% of whom had blood lead levels exceeding 10 µg/L, a threshold considered clinically significant (6).

Beyond its environmental consequences, this situation constitutes a significant public health concern for children, particularly regarding neurological and cognitive development. Even at low concentrations, chronic exposure to heavy metals has been shown to disrupt brain development during critical periods of childhood. These disruptions are associated with impairments in cognitive function, memory, attention, and academic performance (7–9). A growing body of evidence indicates that children exposed to metals such as As and Pb are at increased risk of neurodevelopmental disorders, reduced intelligence quotient (IQ), and learning difficulties (10–13).

Similar environmental health risks have been documented across various regions of Mexico, where elevated concentrations of heavy metals present a persistent public health concern. Research in industrial and mining areas such as Torreón, Hidalgo, Veracruz, San Luis Potosí, and Guerrero has identified high levels of As, Pb, cadmium (Cd), and manganese (Mn) in air, soil, water, and food (14–18). Children living near smelters and mining operations frequently show elevated blood metal levels, which are associated with cognitive impairment, attention deficits, behavioral disturbances, and motor dysfunction. Notably, exposure to Mn and Pb via air and contaminated food has been linked to reduced cognitive performance and, in some cases, lower fertility (19). In regions like San Luis Potosí and Guerrero, Pb and As exposures have been tied to reduced verbal IQ, memory deficits, and poor academic achievement (20–22). These effects are exacerbated by co-exposure to mercury (Hg), as shown in Hidalgo's Molango district, where airborne Mn was inversely associated with cognitive performance (23). This body of evidence highlights the broader national challenge posed by the inadequate regulation of industrial emissions, and the synergistic impact of multiple environmental toxicants on child development.

A growing body of evidence, including studies from Mexico, indicates that early-life exposure to heavy metals and metalloids adversely affects neurodevelopment. Children exposed to air pollution, toxic metals, or pesticides face heightened risks for disorders such as autism spectrum disorder (ASD), attention deficit hyperactivity disorder (ADHD), and cognitive delays (8, 24). Even low-level exposure through food, water, air, or soil can disrupt key neurodevelopmental processes, particularly during sensitive periods (25, 26). The risk increases under combined exposures and socioeconomic stress, which may act synergistically to intensify cognitive and behavioral impairments (21, 27–29).

While the neurotoxic effects of heavy metals are well established, they reflect only one aspect of broader neurodevelopmental risk. Prenatal stress, early-life adversity, and structural inequalities, common in environmentally burdened and underserved communities, further compromise child development by increasing exposure and limiting access to protective resources (30, 31). Additionally, endocrine-disrupting chemicals like bisphenol A, phthalates, Per- and polyfluoroalkyl substances (PFAS), prevalent in low-income settings, disrupt hormonal pathways essential for brain development (32).

Following the 2014 toxic spill from the Buenavista del Cobre mine, children in affected communities have experienced chronic exposure to elevated levels of Pb, As, and other toxicants through contaminated drinking water, soil, and food sources (3, 4). Biomarkers of exposure such as elevated blood Pb and Mn have been associated with impairments in language processing and cognitive performance (5, 10). Recent longitudinal research conducted in rural municipalities of Sonora by Farías et al. (33) further demonstrates that even low-level prenatal exposure to Pb is significantly associated with reduced neurodevelopmental scores during the first year of life, particularly in language domains.

In the context of the SRB, the objective of this study is to evaluate the impact of environmental contamination on neurodevelopment and learning outcomes in children aged 5–13 years living in seven rural and semi-urban communities along the SRB. This population includes children who have grown up in the region during the 11 years following the 2014 toxic spill, in which approximately 40,000 m^3^ of sulfuric acid and copper sulfate leachate from the Buenavista del Cobre mine was released into the SRB, causing long-term ecological and public health concerns. To establish comparative benchmarks, a control group from a rural community without active mining or documented environmental contamination is included. Using a standardized neuropsychological test battery, the study assesses core domains related to attention, executive function, and academic skills. Additionally, it examines how contextual factors such as parenting practices, nutritional status, and prenatal biological risks interact with environmental exposure to influence cognitive development. By integrating cognitive, biological, and psychosocial data, the study aims to provide a multidimensional analysis of the factors shaping learning outcomes in environmentally vulnerable populations and to inform targeted public health and educational interventions.

## Method

### Participants and design

This study employed a cross-sectional design with the inclusion of a control group. A total of 238 children, aged 5–13 years (M = 8.9, SD = 1.9), participated in the assessment. The group exposed to active mining included children from rural communities within the Upper Sonora River Basin, specifically Cananea (*n* = 29), Aconchi (*n* = 52), Baviácora (*n* = 33), Ures (*n* = 31), Banámichi (*n* = 29), San Felipe (*n* = 23), and Huépac (*n* = 18). The selection of these communities was strategically based on their geographic location along the Sonora River stream network, where contamination from the 2014 mining disaster was most concentrated (1, 3). This region has consistently shown elevated concentrations of As, Pb, Cu, Zn, and other toxic metals in surface water, groundwater, soil, and local food sources (2, 4, 5).

In contrast, the control group comprised 23 children from Álamos, Sonora, a rural community with no active mining operations or documented environmental contamination. Álamos shares important climatic, hydrological, and socio-demographic characteristics with the SRB communities but remains geographically and environmentally isolated from the contamination zone. This made it an appropriate control site, allowing for meaningful comparisons between children from exposed and non-exposed populations. As illustrated in Figure 1, Álamos lies outside the contaminated watershed, yet within a rural biosphere reserve system, offering ecological and cultural continuity while serving as a baseline for evaluating neurodevelopmental impacts associated with environmental exposure (see Figure 1).

To ensure comparability across sites, inclusion criteria for the exposed group required that participants reside in communities within the SRB. For the control group, eligibility was restricted to children living in a rural area without active mining operations or documented environmental contamination. Recruitment for both groups was conducted through local schools and community announcements. All participants were assigned a unique identification code to maintain confidentiality. The study received ethical approval from the Bioethics Committee of the Sonora Institute of Technology (Approval No. 218). Written informed consent was obtained from caregivers, and assent was provided by participating children, in accordance with international ethical standards for research involving minors.

The sample size was calculated based on population data from the 2020 Population and Housing Census, published by the Instituto Nacional de Estadística, Geografía e Informática (INEGI). Specifically, we identified the total number of children aged 5–13 years residing in the eight participating municipalities: Cananea, Aconchi, Baviácora, Ures, Banámichi, San Felipe, Huépac, and Álamos. According to census data, this population totaled 3,047 children (see Supplementary Material).

Using this population estimate, we applied a sample size formula for finite populations with a 95% confidence level and a 5% margin of error, resulting in a minimum required sample size of 342 participants to ensure representativeness across all municipalities. Due to logistical and consent-related limitations in some rural sites, the final sample included 238 children, representing approximately 69.6% of the calculated target. Despite the reduced number, we maintained proportional representation from each municipality based on its relative population size.

### Instruments

*The Neuropsychological Battery for the Assessment of Learning Disorders (BANETA)*, developed by Yáñez and Prieto (34), is a comprehensive instrument designed to assess cognitive and learning-related functions in school-aged children. It comprises 41 subtests that evaluate a wide range of domains, including attention, language processing, memory, sensory integration, and academic skills. The battery was originally validated with a representative sample of 425 children aged 7–12 years from Mexico City. Psychometric analyses indicated satisfactory internal consistency, with most subtests showing Cronbach's alpha coefficients exceeding 0.60, supporting its reliability and diagnostic utility. We employed a targeted subset of subtests to evaluate key cognitive domains associated with academic performance and neurodevelopment. The selected subscales included: Attention, Verbal Comprehension, Reading, Writing, Arithmetic, Graphesthesia, Motor Coordination, and Motor Slowness. For the Verbal Comprehension, Reading, Writing, Arithmetic, Graphesthesia, Motor Coordination, and Motor Slowness subscales, the dependent variables analyzed were the number of correct responses and the time in seconds required to complete each task. Specifically for the Attention subscale, three dependent variables were recorded: (1) number of correct responses to target stimuli, (2) number of omission errors (failures to respond to a target stimulus), and (3) number of commission errors (responses to non-target stimuli).

*The Behavior Rating Inventory of Executive Function (BRIEF)* is a validated behavioral questionnaire developed to assess executive functioning in children, typically between 5 and 18 years of age (35). The BRIEF is completed by parents, caregivers, or teachers and consists of 63 items, typically administered in approximately 10–15 min. It assesses behaviors across three core indices of executive functioning: the *Behavioral Regulation Index* (including Self-Monitor and Inhibit), the *Emotional Regulation Index* (comprising Shift and Emotional Control), and the *Cognitive Regulation Index* (encompassing Initiate, Working Memory, Plan/Organize, Organization of Materials, and Task-Monitor). The original validation studies demonstrated excellent internal consistency, with a Cronbach's alpha of 0.95 (35). In addition to its original validation, the BRIEF-P was adapted and validated for use in the Mexican population by García-Anacleto and Salvador-Cruz (36). In this adaptation, the Mexican version demonstrated strong psychometric properties, with a high internal reliability index (Cronbach's *α* = 0.939) and satisfactory factorial validity. The adapted version maintained the original structure, confirming its utility for culturally sensitive evaluations of executive functioning. In this study, the dependent variable was the total score for each subscale of the BRIEF, where higher scores indicate greater executive functioning difficulties, and lower scores reflect better executive self-regulation.

*The Parenting Practices Inventory (IPC),* developed by López (37), is a self-report, pencil-and-paper questionnaire completed by parents or primary caregivers. It is designed to assess parenting behaviors related to discipline strategies and the promotion of affection within the family context. The IPC consists of 40 closed-ended items rated on a 7-point Likert scale ranging from 0 (“Never”) to 6 (“Always”), with an average completion time of approximately 20 min. Exploratory factor analysis identified six subscales: Punishment, Material Rewards, Social Interaction, Rules, Social Rewards, and Limits. The instrument demonstrated high internal consistency (Cronbach's *α* = 0.89) and accounted for 61.85% of the total explained variance.

In the context of this study, the IPC serves as a valuable tool for identifying specific parenting styles that may influence children's cognitive performance and behavioral regulation. Harsh or inconsistent disciplinary practices, as reflected in the Punishment subscale, may be associated with greater executive functioning difficulties or behavioral problems, while more supportive practices such as rule setting and positive reinforcement may promote better developmental outcomes. The dependent variables analyzed were the total scores for each subscale, where higher scores indicate a greater frequency and presence of the corresponding parenting practices.

*The Mini Nutritional Assessment (MNA)* is a validated, non-invasive screening tool traditionally designed to evaluate the nutritional status of older adults. However, recent research supports its adaptation for pediatric use, particularly in clinical populations with neurodevelopmental or chronic health conditions. For instance, Grot et al. (38) applied a modified version of the MNA in children and adolescents with neurodevelopmental disorders, demonstrating its utility for identifying nutritional risk in younger populations. Similarly, the Multifactorial Impairment of Nutrition in Infants and Children (MINI) composite, which includes MNA-based parameters, has been validated as a marker of compromised nutritional status in pediatric patients undergoing hemodialysis (39). These applications highlight the growing relevance of the MNA framework beyond geriatric settings. In the present study, we used the short-form version (MNA-SF), consisting of six items assessing food intake, recent weight loss, mobility, psychological stress or acute disease, neuropsychological problems, and BMI. The MNA-SF has shown good agreement with the original 18-item tool and provides a rapid, reliable alternative when comprehensive nutritional assessments are not feasible (50). The dependent variable in this study is the total score on the MNA-SF, where higher scores indicate better nutritional status.

*The Child Development Assessment (Evaluación del Desarrollo Infantil EDI)* is a validated, population-specific developmental screening tool designed to detect early developmental problems in Mexican children under five years of age (40). The instrument evaluates developmental domains such as fine and gross motor skills, language, social interaction, and adaptive behavior. It is organized according to age-specific bands and includes both caregiver-reported items and direct observations, utilizing a traffic-light classification system (green, yellow, red) to indicate normal development, potential developmental delay, or probable delay requiring further assessment. The EDI has demonstrated robust psychometric properties. In the national validation study, the instrument showed sensitivity of 81.5%, specificity of 73.3%, and positive predictive value of 62.5%, indicating adequate accuracy for use in community and primary care settings. Additionally, internal consistency across developmental domains was acceptable, and inter-rater reliability was reported as high, supporting the tool's reliability in diverse contexts. In the present study, a seven-question screening module derived from the EDI was used to assess biological risk factors primarily associated with prenatal and perinatal periods. The dependent variable in this study was the cumulative score from the seven EDI-based screening items, where higher scores reflect increased biological risk exposure potentially associated with adverse neurodevelopmental outcomes.

### Participant recruitment and assessment procedures

The assessments were administered by a team of six psychologists who completed a three-month intensive training program focused on the standardized application of the neuropsychological battery and associated psychological instruments. The training included supervised practice, inter-rater calibration exercises to ensure scoring reliability, and in-depth review of the cognitive domains assessed by the battery, including attention, memory, phonological processing, and reading skills. Data collection was carried out between August 2023 and January 2025, allowing for the evaluation of children exposed to chronic environmental conditions in the years following the 2014 Sonora River toxic spill.

Participant recruitment was initiated through coordinated outreach to elementary school principals in each target community. To enhance study visibility and community engagement, we also collaborated with local leaders in health and education, including parent associations and health promoters. Informational brochures outlining the study's objectives, procedures, and ethical safeguards were distributed both in schools and through these community channels. This preliminary engagement played a crucial role in building trust and ensuring informed, voluntary participation.

Once school participation was confirmed, families were offered the choice to complete the evaluation either in their homes or at designated school facilities. For school-based assessments, quiet and air-conditioned classrooms were arranged to minimize distractions and maintain consistent testing conditions. All assessments were conducted in the presence of parents or legal guardians, who provided written informed consent prior to data collection. Children provided assent in accordance with ethical research guidelines for minors. During the assessment, one psychologist worked with the child while another simultaneously interviewed the caregiver.

All assessments were conducted following a standardized protocol to ensure consistency across both the exposed (mining-region) and control (non-exposed) groups. Children individually completed a battery of neuropsychological tasks derived from the BANETA. Simultaneously, caregivers completed the instruments: (1) the IPC; (2) the MNA-SF; (3) the BRIEF; and (4) the EDI.

### Data analysis

For the analysis of IPC and BRIEF data, *z*-scores were calculated based on the mean and standard deviation of the Álamos control group. A *z*-score of 0 indicates performance equivalent to the control group mean, whereas positive and negative values reflect deviations above or below this reference point, respectively. In contrast, *z*-scores for the BANETA tests were calculated using the standardized norms provided in the test manual, based on a reference sample of children from Mexico City (CDMX). The manual offers age-specific normative data for 7, 8, 9–10, and 11–13-year-olds. For analytical consistency, children under the age of 7 were compared to the 7-year-old normative group, while those over 13 were compared to the 11–13-year-old reference group.

To determine the appropriate statistical tests, Levene's test and the Shapiro–Wilk test were conducted to assess the assumptions of homogeneity of variances and normality, respectively. One-way ANOVA was used to compare age, weight, and height across communities. For the IPC, BRIEF, and BANETA variables, the non-parametric Kruskal–Wallis test was applied. *Post hoc* comparisons were performed using Dunn's test, and effect sizes were calculated with an adjusted non-parametric version of *η*^2^. Additionally, a correlation matrix was computed to examine associations among key study variables, including biological risk factors (assessed via the EDI), nutritional status (MNA-SF), and parenting practices (IPC), in relation to executive functioning (BRIEF-P) and learning-related cognitive domains (BANETA subscales).

To examine the contribution of individual predictors to the probability of exposure, a logistic regression model was fitted using the scikit-learn library in Python. The model was trained to classify participants into exposed and non-exposed groups based on previously described *z*-scores. The predictors included child weight, child height, MNA, Biological risks, BRIEF indices, IPC subscales, and BANETA subtests. Given the class imbalance in the sample (SRB group vs. Álamos control group), the Synthetic Minority Over-sampling Technique (SMOTE) was applied to generate a balanced dataset during model training. To assess model performance and reduce the risk of overfitting, a 5-fold stratified cross-validation was employed. Model evaluation included confusion matrices, classification metrics (precision, recall, F1-score), and the extraction of regression coefficients. The final model was retrained on the fully balanced dataset to obtain interpretable coefficients (B coefficients, expressed in log-odds), which indicate both the direction and magnitude of each predictor's contribution to the classification. A positive B value denotes a higher log-odds of belonging to the exposed group, while a negative B value indicates a lower log-odds. Additionally, the exponential transformations of these coefficients (odds ratios) were computed to express the same associations on a multiplicative scale: OR values greater than 1 signify increased odds of exposure, whereas OR values below 1 indicate a reduced likelihood of belonging to the exposed group.

All analyses were conducted using JASP version 18.0.0 and Python. The dataset is publicly available, with all identifying information removed to ensure participant confidentiality.

## Results

Children's age, weight, and height were comparable across the participating communities (Table 1). On average, children weighed 35.5 kg (SD = 13.5), and measured 136.3 cm (SD = 14.3). ANOVA tests revealed no statistically significant differences in age, [*F*(7, 230) = 1.65, *p* = 0.123, *η*^2^ = 0.05], weight [*F*(7, 158) = 1.30, *p* = 0.255, *η*^2^ = 0.05] or height [*F*(7, 149) = 0.68, *p* = 0.691, *η*^2^ = 0.03].

**Table 1:** Descriptive values of age, weight, height, MNA scores, and biological risks for the control group (Álamos) and communities along the Sonora river.

Variable	Álamos (*n* = 23)			Cananea (*n* = 29)			Banámichi (*n* = 29)			Huépac (*n* = 18)			San Felipe (*n* = 23)			Aconchi (*n* = 52)			Baviácora (*n* = 33)			Ures (*n* = 31)			ANOVA		
	*x¯*	SD	Med	*x¯*	SD	Med	*x¯*	SD	Med	*x¯*	SD	Med	*x¯*	SD	Med	*x¯*	SD	Med	*x¯*	SD	Med	*x¯*	SD	Med	F	p	*η* ^2^
Age (years)	9.26	2.03	10.00	8.97	1.86	9.00	9.21	1.90	10.00	7.83	1.62	7.50	9.35	1.99	9.00	9.14	1.95	9.00	8.76	2.06	8.00	8.39	1.82	8.00	1.65	0.12	0.05
Weight (kg)	33.24	11.55	34.50	35.67	11.63	32.00	37.14	12.01	38.50	28.82	12.01	27.00	39.00	17.24	36.00	33.95	12.50	29.00	39.48	17.17	36.00	38.63	14.63	37.50	1.30	0.26	0.05
Height (cm)	138.85	11.92	137.50	138.86	17.61	144.50	137.92	14.21	140.00	131.29	8.96	130.00	135.94	24.09	139.00	134.90	12.62	132.00	139.29	12.22	140.00	134.71	12.42	132.00	0.68	0.69	0.03
																									Kruskal–Wallis		
MNA	−0.01	0.81	0.03	0.38	0.80	0.40	0.06	1.01	0.03	0.11	0.93	0.12	0.08	0.93	0.09	−0.30	1.41	−0.13	−0.03	1.08	−0.09	0.06	0.88	0.06	20.62	**0.004**	0.06
B. Risks	0.46	1.11	0.05	0.18	1.00	0.05	−0.08	0.92	−0.42	−0.39	0.65	−0.65	−0.33	0.52	−0.42	0.04	0.98	−0.42	0.01	1.44	−0.42	−0.04	0.77	0.05	13.00	**0.07**	0.04

Bold values indicate statistically significant differences among the groups.

**Figure 2:**
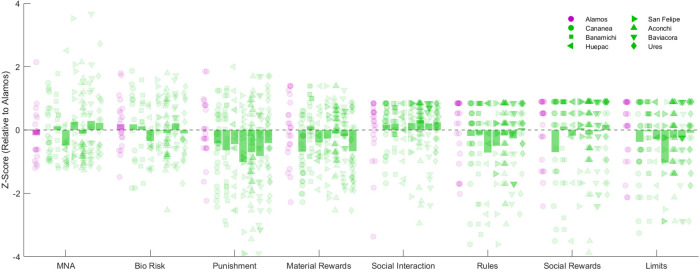
IPC *z*-scores relative to the Álamos control group, and *z*-scores for MNA and biological risk indicators. Purple dots represent individual participants from Álamos, while green dots represent participants from communities along the Sonora River. Bars indicate the average scores for each group. A score of 0 reflects performance similar to the mean of the Álamos group, while positive or negative values indicate higher or lower scores, respectively, relative to the Álamos group mean.

*The MNA* scores for the Álamos control group were similar to those of the SRB communities (*M* = −0.01, *SD* = 0.81). Among the SRB communities, the highest score was observed in Cananea (*M* = 0.38, *SD* = 0.80), whereas the lowest score was found in Aconchi (*M* = −0.30, *SD* = 1.41). A Kruskal–Wallis test indicated statistically significant differences across communities [*H*(7) = 20.62, *p* = 0.004, *η*^2^ = 0.06]. *Post hoc* Dunn's tests revealed a significant difference between Álamos and Cananea (*p* < 0.05). Similarly, for biological risks, the control group showed comparable scores to several SRB communities (*M* = 0.46, *SD* = 1.11). Among these communities, the highest biological risk score was observed in Cananea (*M* = 0.18, *SD* = 1.00), while the lowest was recorded in Huepac (*M* = −0.39, *SD* = 0.65). However, a Kruskal–Wallis test indicated no statistically significant differences across communities [*H*(7) = 13.00, *p* = 0.072, *η*^2^ = 0.04] (Table 1, Figure 2).

Regarding *IPC Punishment* (Table 2), SRB communities scored lower than the Álamos control group. The lowest scores were observed in San Felipe (*M* = −1.01, *SD* = 1.59), while the highest were found in Ures (*M* = −0.41, *SD* = 1.36). A Kruskal–Wallis test revealed no statistically significant differences across communities [*H*(7) = 6.67, *p* = 0.464, *η*^2^ = 0.02]. In terms of *Material rewards*, SRB communities below the control group. The lowest scores were recorded in Ures (*M* = −0.67, *SD* = 0.98), and the highest in Banámichi (*M* = 0.11, *SD* = 0.84). A Kruskal–Wallis test indicated statistically significant differences among communities [*H*(7) = 19.68, *p* = 0.006, *η*^2^ = 0.06]. *Post hoc* Dunn's tests identified significant differences between Álamos and two Sonora River communities (Cananea and Baviácora; *p* < 0.05). Regarding *Social interaction*, the SRB communities performed similarly to Álamos. Aconchi showed the highest scores (*M* = 0.32, *SD* = 1.44), while the lowest were observed in Huépac (*M* = 0.01, *SD* = 0.77). No statistically significant differences were detected [*H*(7) = 4.46, *p* = 0.725, *η*^2^ = 0.01]. For *Rules*, scores across SRB communities did not differ significantly from those of Álamos. Ures had the highest scores (*M* = 0.06, *SD* = 0.88), and Huépac the lowest (*M* = −0.71, *SD* = 2.28). The Kruskal–Wallis test confirmed no significant differences [*H*(7) = 6.31, *p* = 0.504, *η*^2^ = 0.02]. Regarding *Social rewards*, no significant differences were observed among communities [*H*(7) = 3.67, *p* = 0.817, *η*^2^ = 0.01]. Ures obtained the highest scores (*M* = 0.16, *SD* = 0.95), while Cananea had the lowest (*M* = −0.70, *SD* = 2.42). Finally, in terms of *Limits subscale*, SRB communities showed comparable scores to Álamos. Aconchi presented the highest scores (*M* = −0.25, *SD* = 1.13), and San Felipe the lowest (*M* = −1.03*, SD* = 1.56). The Kruskal–Wallis test did not reveal statistically significant differences [*H*(7) = 9.63, *p* = 0.211, *η*^2^ = 0.03].

**Table 2:** Descriptive values of IPC scores (punishment, tangible rewards, social interaction, rule-following, social rewards, and limits) for the control group (Álamos) and communities along the Sonora river.

IPC	Álamos (*n* = 23)			Cananea (*n* = 29)			Banámichi (*n* = 29)			Huépac (*n* = 18)			San Felipe (*n* = 23)			Aconchi (*n* = 52)			Baviácora (*n* = 33)			Ures (*n* = 31)			Kruskal–Wallis		
*N*	*x¯*	SD	Med	*x¯*	SD	Med	*x¯*	SD	Med	*x¯*	SD	Med	*x¯*	SD	Med	*x¯*	SD	Med	*x¯*	SD	Med	*x¯*	SD	Med	H	p	n2
Punishment	0.00	1.00	−0.12	−0.42	1.01	−0.42	−0.64	1.45	−0.50	−0.45	1.31	−0.42	−1.01	1.59	−0.88	−0.70	1.31	−0.88	−0.82	1.61	−0.57	−0.41	1.36	−0.272	6.67	0.464	0.02
Material Rewards	0.00	1.00	0.08	−0.68	0.89	−0.71	0.11	0.84	0.08	−0.39	0.82	−0.58	−0.27	0.97	−0.31	−0.09	0.84	0.08	−0.20	0.88	−0.05	−0.67	0.98	−0.968	19.68	**0.006**	0.06
Social Interaction	0.00	1.00	0.28	0.16	0.72	0.28	0.19	0.54	0.28	0.01	0.77	0.21	0.21	0.72	0.42	0.32	1.44	0.42	0.20	0.90	0.70	0.24	0.51	0.28	4.46	0.725	0.01
Rules	0.00	1.00	0.53	−0.19	1.10	0.21	−0.15	1.25	0.21	−0.71	2.28	0.05	−0.50	1.14	−0.11	−0.15	0.98	0.21	−0.27	0.99	−0.11	0.06	0.88	0.21	6.31	0.504	0.02
Social Rewards	0.00	1.00	0.53	−0.70	2.42	0.16	0.11	1.19	0.53	−0.18	1.49	0.34	0.06	0.95	0.16	−0.14	1.31	0.16	−0.04	1.08	0.16	0.16	0.95	0.89	3.67	0.817	0.01
Limits	0.00	1.00	0.13	−0.38	1.24	−0.62	−0.04	0.96	0.13	−0.29	1.28	−0.06	−1.03	1.56	−0.25	−0.25	1.13	0.13	−0.23	1.02	−0.25	−0.09	1.00	0.13	9.63	0.211	0.03

Bold values indicate statistically significant differences among the groups.

**Figure 3:**
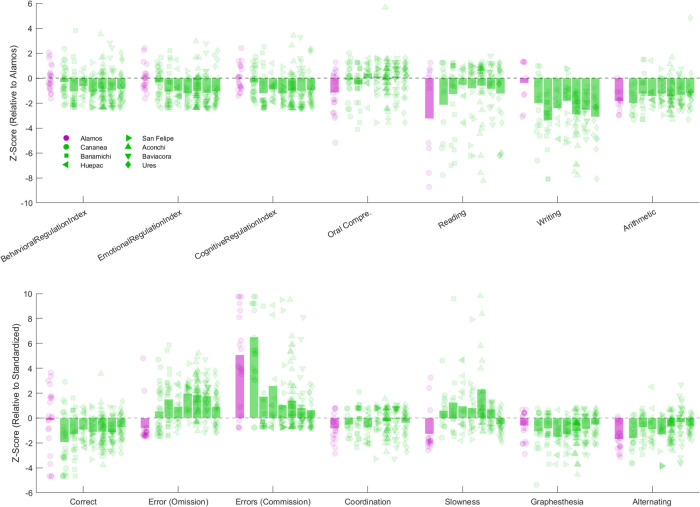
BRIEF and BANETA *z*-scores relative to scores from the Álamos control group. Purple dots represent individual participants from Álamos, while green dots represent participants from communities along the Sonora River. Bars indicate the average scores for each group. A score of 0 reflects performance similar to the Álamos group mean on BRIEF subscales, while a score of 0 on BANETA reflects performance similar to the Mexico City (CDMX) standardization sample. Positive or negative values indicate higher or lower scores, respectively.

Figure 3 shows the BRIEF and BANETA *z*-scores relative to the Álamos control group. In the BRIEF behavioral regulation index (BRI), SRB communities scored lower than control group. The lowest mean score was observed in Banámichi (*M* = −1.07, *SD* = 1.48), while the highest was in Cananea (*M* = −0.32, *SD* = 1.15). A Kruskal–Wallis test revealed statistically significant differences across communities [*H*(7) = 25.77, *p* < 0.001, *η*^2^ = 0.08]. *Post hoc* Dunn's tests indicated significant differences between control group and five Sonora River communities (Aconchi, Banámichi, Baviácora, San Felipe, and Ures; *p* < 0.01). For emotional regulation (ERI), a similar pattern was observed, SRB communities scored lower than control group. Banámichi again presented the lowest mean score (*M* = −1.00, *SD* = 1.46), whereas Cananea had the highest (*M* = −0.26, *SD* = 1.15). A Kruskal–Wallis test indicated statistically significant differences across communities [*H*(7) = 32.47, *p* < 0.001, *η*^2^ = 0.10]. *Post hoc* Dunn's tests revealed significant differences between Álamos and five communities (Banámichi, Baviácora, Huepac, San Felipe, and Ures; *p* < 0.01). For cognitive regulation (CRI), SRB communities scored lower than control group. Banámichi showed the lowest mean score (*M* = −1.21, *SD* = 1.40), and Cananea the highest (*M* = −0.34, *SD* = 1.09). A Kruskal–Wallis test found statistically significant differences across communities [*H*(7) = 30.62, *p* < 0.001, *η*^2^ = 0.09]. *Post hoc* Dunn's tests indicated significant differences between Álamos and five communities (Aconchi, Banámichi, Baviácora, San Felipe, and Ures; *p* < 0.005).

The BANETA Attention subscale includes three dependent variables: (1) the number of correct responses to target stimuli, (2) the number of omission errors (failures to respond to target stimuli), and (3) the number of commission errors (responses to non-target stimuli). Together, these measures offer a comprehensive evaluation of attentional control, response inhibition, and vigilance, cognitive functions that are particularly susceptible to disruption by environmental neurotoxicants. Regarding correct responses (Table 3), SRB communities scored lower compared to the Álamos control group (*M* = −0.16, *SD* = 2.64). Among the SRB communities, the highest score was observed in Ures (*M* = −0.73, *SD* = 0.96), whereas the lowest score was observed in Cananea (*M* = −1.94, *SD* = 2.07). A Kruskal–Wallis test indicated statistically significant differences across communities [*H*(7) = 15.79, *p* = 0.027, *η*^2^ = 0.05]. *Post hoc* Dunn's tests revealed statistically significant differences between Álamos and four SRB communities (Cananea, Banámichi, Baviácora, and San Felipe; *p* < 0.05). Regarding omission errors in attention, SRB communities scored higher compared to the Álamos control group (*M* = −0.80, *SD* = 1.46). Within the SRB communities, the highest scores were observed in San Felipe (*M* = 1.96, *SD* = 1.07), whereas the lowest scores were observed in Cananea (*M* = 0.53, *SD* = 1.81). A Kruskal–Wallis test indicated statistically significant differences across communities [*H*(7) = 56.63, *p* < 0.001, *η*^2^ = 0.16]. *Post hoc* Dunn's tests revealed statistically significant differences between Álamos and all SRB communities (Aconchi, Banámichi, Baviácora, Cananea, Huépac, San Felipe, and Ures; *p* < 0.005). Regarding commission errors, Álamos (*M* = 5.07, *SD* = 4.03) had higher scores compared to most SRB communities. The community with the highest commission errors was Cananea (*M* = 6.49, *SD* = 3.43), whereas the lowest score was observed in Banámichi (*M* = 1.72, *SD* = 5.40). A Kruskal–Wallis test indicated statistically significant differences across communities [*H*(7) = 53.65, *p* < 0.001, *η*^2^ = 0.16]. *Post hoc* Dunn's tests revealed statistically significant differences between Álamos and five SRB communities (Aconchi, Baviácora, Banámichi, Cananea, and Ures; *p* < 0.05).

**Table 3:** BRIEF and BANETA *z*-scores relative to scores from the Álamos control group.

BRIEF	Álamos (*n* = 23)			Cananea (*n* = 29)			Banámichi (*n* = 29)			Huépac (*n* = 18)			San Felipe (*n* = 23)			Aconchi (*n* = 52)			Baviácora (*n* = 33)			Ures (*n* = 31)			Kruskal–Wallis		
*N*	*x¯*	SD	Med	*x¯*	SD	Med	*x¯*	SD	Med	*x¯*	SD	Med	*x¯*	SD	Med	*x¯*	SD	Med	*x¯*	SD	Med	*x¯*	SD	Med	H	*p*	*n* ^2^
Behavioral Regulation (BRI)	0.00	1.00	−0.11	−0.32	1.15	−0.57	−1.07	1.48	−1.65	−0.63	0.78	−0.63	−1.09	0.94	−1.19	−0.86	1.28	−0.96	−1.00	1.42	−1.46	−0.86	1.15	−1.146	25.77	**<**.**001**	0.08
Emotional Regulation (ERI)	0.00	1.00	−0.08	−0.26	1.15	−0.26	−1.00	1.46	−1.47	−0.84	0.91	−0.62	−0.85	0.83	−1.14	−0.76	1.27	−1.02	−0.96	1.42	−1.47	−0.87	1.21	−1.474	32.47	**<**.**001**	0.1
Cognitive Regulation (CRI)	0.00	1.00	−0.09	−0.34	1.09	−0.40	−1.21	1.40	−1.80	−0.89	0.81	−0.83	−1.22	0.89	−1.39	−1.02	1.25	−1.04	−1.02	1.49	−1.48	−0.96	1.27	−1.183	30.62	**<**.**001**	0.09
BANETA																											
Attention Correct Responses	−0.16	2.64	0.07	−1.94	2.07	−2.07	−1.28	1.59	−1.22	−0.93	1.12	−1.15	−1.10	1.03	−1.25	−1.09	1.29	−1.19	−1.15	0.98	−1.17	−0.73	0.96	−0.788	15.792	**0**.**027**	0.05
Omission Errors (Attention)	−0.80	1.46	−1.27	0.53	1.81	−0.11	1.50	1.94	1.49	0.90	1.38	0.71	1.96	1.07	2.36	1.83	1.48	1.84	1.74	1.33	1.84	0.88	1.35	0.902	56.63	**<**.**001**	0.16
Commission Errors	5.07	4.03	4.85	6.49	3.43	5.44	1.72	5.40	−0.32	2.58	4.58	0.34	1.04	2.84	0.00	1.39	2.33	0.76	0.80	2.01	0.16	0.63	2.64	−0.077	53.648	**<**.**001**	0.16
Oral Comprehension	−1.16	1.68	−1.03	−0.14	1.38	0.11	−0.48	1.44	−0.34	0.37	0.94	0.60	0.12	1.19	0.28	−0.09	1.47	0.01	0.10	1.11	0.41	0.01	1.24	0.199	14.314	**0**.**046**	0.04
Reading	−3.24	4.78	−0.58	−2.14	3.98	−0.43	−1.28	2.76	−0.19	−0.54	1.56	0.03	−0.82	2.54	0.40	−0.61	2.34	0.48	−0.87	3.09	−0.15	−1.24	2.57	−0.31	4.898	0.672	0.01
Writing	−0.39	1.85	0.02	−2.01	2.26	−1.79	−3.39	1.94	−2.82	−2.41	1.52	−2.18	−1.83	1.86	−1.35	−2.88	1.86	−2.23	−2.54	1.20	−2.42	−3.09	1.77	−2.343	20.041	**0**.**005**	0.06
arithmetic problems	−1.84	0.84	−1.80	−2.00	0.97	−2.13	−1.25	1.43	−1.36	−1.44	0.96	−1.74	−1.24	0.96	−1.44	−1.46	1.12	−1.57	−1.35	0.96	−1.65	−1.19	1.52	−1.288	7.089	0.42	0.02
Motor Coordination	−0.83	1.10	−0.93	−0.52	0.80	−0.62	−0.04	1.13	−0.04	−0.74	1.03	−0.70	−0.02	1.01	0.13	−0.27	0.99	−0.04	−0.10	1.20	0.53	−0.38	1.04	−0.044	15.086	**0**.**035**	0.05
Motor Slowing	−1.27	1.64	−1.85	0.58	1.42	0.34	1.25	3.20	0.33	0.95	1.97	0.55	0.81	2.19	0.48	2.30	4.37	0.64	0.68	3.06	0.15	−0.49	0.74	−0.495	43.291	**<**.**001**	0.13
Graphesthesia	−0.61	1.11	−0.41	−1.06	1.54	−1.02	−0.83	1.12	−0.92	−1.51	1.11	−1.46	−1.30	1.02	−1.24	−1.07	1.27	−0.84	−0.86	1.14	−0.84	−0.52	0.90	−0.305	12.94	0.074	0.04
Alternation	−1.68	0.98	−1.78	−1.60	1.22	−1.66	−0.74	1.04	−0.52	−0.89	1.22	−1.00	−1.32	1.44	−0.89	−0.65	1.78	−0.63	−0.33	1.49	−0.17	−0.61	0.61	−0.63	25.108	**<**.**001**	0.07

Bold values indicate statistically significant differences among the groups.

In the domain of oral comprehension, SRB communities scored higher compared to the Álamos control group (*M* = −1.16, *SD* = 1.68). The community with the highest scores was Huépac (*M* = 0.37, *SD* = 0.94), whereas the lowest score among SRB communities was observed in Banámichi (*M* = −0.48, *SD* = 1.44). A Kruskal–Wallis test indicated statistically significant differences across communities [*H*(7) = 14.31, *p* = 0.046, *η*^2^ = 0.04]. *Post hoc* Dunn's tests revealed statistically significant differences between Álamos and six SRB communities (Aconchi, Baviácora, Banámichi, Cananea, Huépac, and Ures; *p* < 0.05). Regarding reading, SRB communities scored higher compared to the Álamos control group (*M* = −3.24, *SD* = 4.78). The community with the highest reading scores was Aconchi (*M* = −0.61, *SD* = 2.34), whereas the lowest score was observed in Cananea (*M* = −2.14, *SD* = 3.98). A Kruskal–Wallis test indicated no statistically significant differences across communities [*H*(7) = 4.90, *p* = 0.672, *η*^2^ = 0.01]. In terms of writing, all SRB communities scored lower compared to the Álamos control group (*M* = −0.39, *SD* = 1.85). The community with the highest mean score among SRB communities was San Felipe (*M* = −1.83, *SD* = 1.86), whereas the lowest score was observed in Banámichi (*M* = −3.39, *SD* = 1.94). A Kruskal–Wallis test indicated statistically significant differences across communities [*H*(7) = 20.04, *p* = 0.005, *η*^2^ = 0.06]. *Post hoc* Dunn's tests revealed statistically significant differences between Álamos and five Sonora River communities (Aconchi, Banámichi, Baviácora, Cananea, and Huépac; *p* < 0.05). Regarding arithmetic problems, SRB communities scored similarly compared to the Álamos control group (*M* = −1.84, *SD* = 0.84). A Kruskal–Wallis test indicated no statistically significant differences across communities [*H*(7) = 7.089, *p* = 0.420, *η*^2^ = 0.02]. Within SRB communities, the highest score was observed in Ures (*M* = −1.19, *SD* = 1.52), whereas the lowest score was observed in Cananea (*M* = −2.00, *SD* = 0.97).

In the domain of motor coordination, SRB communities scored higher compared to the Álamos control group (*M* = −0.83, *SD* = 1.10). The highest scores among the Sonora River communities were observed in Baviácora (*M* = −0.10, *SD* = 1.20), whereas the lowest scores were observed in Huépac (*M* = −0.74, *SD* = 1.03). A Kruskal–Wallis test indicated statistically significant differences across communities [*H*(7) = 15.09, *p* = 0.035, *η*^2^ = 0.05]. *Post hoc* Dunn's tests revealed statistically significant differences between Álamos and three SRB communities (Banámichi, Baviácora, and Huépac; *p* < 0.05). Regarding motor Slowing, SRB communities scored higher compared to the Álamos control group (*M* = −1.27, *SD* = 1.64). The community with the highest scores was Aconchi (*M* = 2.30, *SD* = 4.37), whereas the lowest score was observed in Ures (*M* = −0.49, *SD* = 0.74). A Kruskal–Wallis test indicated statistically significant differences across communities [*H*(7) = 43.29, *p* < 0.001, *η*^2^ = 0.13]. *Post hoc* Dunn's tests revealed statistically significant differences between Álamos and six Sonora River communities (Aconchi, Banámichi, Baviácora, Cananea, Huépac, and San Felipe; *p* < 0.05). With respect to graphesthesia, SRB communities and Álamos scored similarly. Álamos (*M* = −0.61, *SD* = 1.11) had an intermediate performance compared to the river communities. The community with the highest scores was Ures (*M* = −0.52, *SD* = 0.90), whereas the lowest scores were observed in Huépac (*M* = −1.51, *SD* = 1.11). A Kruskal–Wallis test indicated no statistically significant differences across communities [*H*(7) = 12.94, *p* = 0.074, *η*^2^ = 0.04]. Finally, regarding alternating, SRB communities scored higher compared to the Álamos control group (*M* = −1.68, *SD* = 0.98). The community with the highest motor coordination scores was Baviácora (*M* = −0.33, *SD* = 1.49), whereas the lowest score was observed in Cananea (*M* = −1.60, *SD* = 1.22). A Kruskal–Wallis test indicated statistically significant differences across communities [*H*(7) = 25.11, *p* < 0.001, *η*^2^ = 0.07]. *Post hoc* Dunn's tests revealed statistically significant differences between Álamos and four SRB communities (Aconchi, Baviácora, Banámichi, and Ures; *p* < 0.01) (See Figure 3).

**Figure 4:**
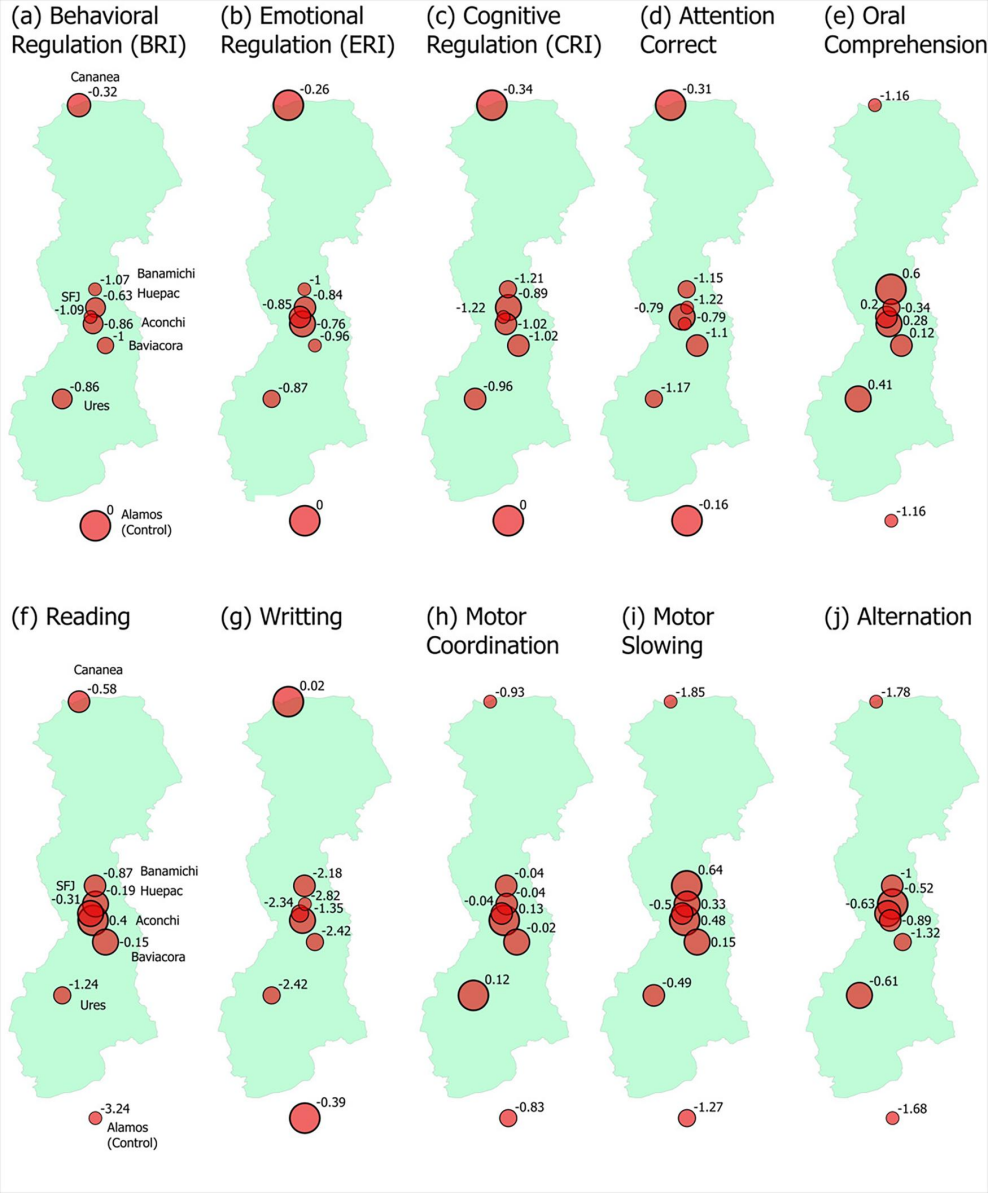
Spatial distribution of BANETA and BRIEF *z*-scores across the Sonora river basin. Larger points represent higher *z*-score magnitudes (regardless of direction). For illustrative purposes, the control group from Álamos is positioned at the southern end of the map, although its actual geographic location is further southwest.

Figure 4 shows a complementary spatial representation of the findings previously reported. It depicts the geographical locations of the seven SRB communities, along with the Álamos control group. To aid in the interpretation of regional patterns in neurodevelopmental outcomes, the figure also presents the spatial distribution of *z*-scores for the BRIEF and BANETA subscales.

To examine the associations among executive functioning, academic skills, attention performance, and contextual variables, Pearson correlation analyses were conducted using *z*-scores for all key measures (Table 4). The Mini Nutritional Assessment was positively associated with biological risks (*p* < 0.01) and with the BRIEF subscales of Behavioral Regulation, Emotional Regulation, and Cognitive Regulation (*p* < 0.001), while showing a negative correlation with the BANETA graphesthesia subtest (*p* < 0.05). Similarly, biological risks were positively correlated with the BRIEF subscales of Behavioral Regulation, Emotional Regulation, and Cognitive Regulation (*p* < 0.05). Regarding the IPC, punishment was the only dimension positively associated with all BRIEF subscales (*p* < 0.001) and also showed a positive correlation with the “limits” subscale. In turn, the “limits” subscale was positively correlated with other IPC dimensions, including material rewards, rules, and social rewards (*p* < 0.05). Among BANETA outcomes, only material rewards were negatively associated with commission errors (*p* < 0.05), while social rewards were positively correlated with motor coordination (*p* < 0.01). Regarding BANETA outcomes, oral comprehension was positively associated with reading, arithmetic, correct responses on the attention task, motor coordination, and graphesthesia (*p* < 0.05). Similarly, reading scores were positively correlated with writing, motor coordination, and graphesthesia (*p* < 0.05). Finally, the number of correct responses on the attention task was negatively correlated with both omission and commission errors, and positively associated with motor speed and alternating motor coordination (*p* < 0.05).

**Table 4:** BRIEF and BANETA *z*-scores relative to scores from the Álamos control group.

Variables	1	2	3	4	5	6	7	8	9	10	11	12	13	14	15	16	17	18	19	20	21
1. MNA	—																				
2. Bio Risk	0.185**	—																			
3. Punishment	0.083	0.025	—																		
4. Material Rew.	−0.041	−0.01	0.132*	—																	
5. Social Intera.	0.074	0.115	−0.111	0.126	—																
6. Rules	0.062	0.055	0.092	0.092	0.393***	—															
7. Social Rew.	−0.015	0.079	−0.032	0.297***	0.326***	0.358***	—														
8. Limits	−0.036	−0.007	0.337***	0.155*	0.252***	0.556***	0.224***	—													
9. Behavioral Reg	0.266***	0.187**	0.301***	−0.004	−0.088	0.032	−0.063	−0.023	—												
10. Emotional Reg	0.254***	0.163*	0.381***	0.051	−0.09	0.056	0.002	0.016	0.843***	—											
11. Cognitive Reg	0.252***	0.189**	0.347***	0.071	−0.066	0.054	−0.035	0.024	0.876***	0.915***	—										
12. Oral Compre	−0.007	0.053	−0.143*	−0.073	0.099	0.004	0.118	−0.006	−0.206**	−0.154*	−0.158*	—									
13. Reading	−0.058	−0.004	−0.159*	0.003	0.048	0.031	−0.113	0.039	−0.207**	−0.265***	−0.264***	0.174*	—								
14. Writing	−0.023	0.012	0.038	−0.048	−0.041	0.177*	−0.112	0.025	−0.183*	−0.208**	−0.217**	−0.038	0.29**	—							
15. Arithmetic	0.012	0.086	−0.084	−0.067	−0.025	0.056	0.095	−0.068	−0.189*	−0.198*	−0.195*	0.187*	0.184*	0.202*	—						
16. Correct	0.027	0.105	−0.038	−0.016	−0.05	0.03	0.044	−0.042	−0.096	−0.067	−0.089	0.187**	0.093	0.123	0.369***	—					
17. Error (Omi)	0.012	−0.049	0.001	0.044	0.024	0.021	−0.043	0.043	−0.021	−0.029	−0.011	0.084	0.03	−0.159*	−0.193*	−0.36***	—				
18. Errors (Com)	−89.31	−0.005	0.067	−0.153*	0.047	0.015	−0.063	0.097	0.12	0.106	0.112	0.008	−0.148	0.038	−0.205**	−0.213**	−0.168*	—			
19. Coordination	−0.038	−0.087	−0.187**	−0.003	0.098	0.052	0.183**	−0.013	−0.14*	−0.106	−0.125	0.241***	0.248***	0.126	0.193*	0.001	0.074	−0.105	—		
20. Slowness	0.045	0.037	−0.017	0.07	0.024	−0.011	0.029	−0.084	0.076	0.059	0.077	−0.177*	−0.002	−0.167	−0.198*	−0.181*	0.115	−0.084	−0.006	—	
21. Graphesthesia	−0.138*	−0.024	−0.023	0.026	0.002	0.112	0.027	0.048	−0.103	−0.163*	−0.136	0.167*	0.181*	0.189*	0.063	0.122	−0.011	0.002	0.246***	−0.244**	—
22. Alternating	−0.003	−0.051	−0.174*	−0.015	−0.023	−0.075	−0.068	−0.069	−0.111	−0.168*	−0.132	0.117	0.249**	0.015	0.336***	0.216**	−0.054	−0.26***	0.051	0.038	−0.054

Bold values indicate statistically significant differences among the groups.

* *p* < 0.05.

** *p* < 0.01.

*** *p* < 0.001.

**Figure 5:**
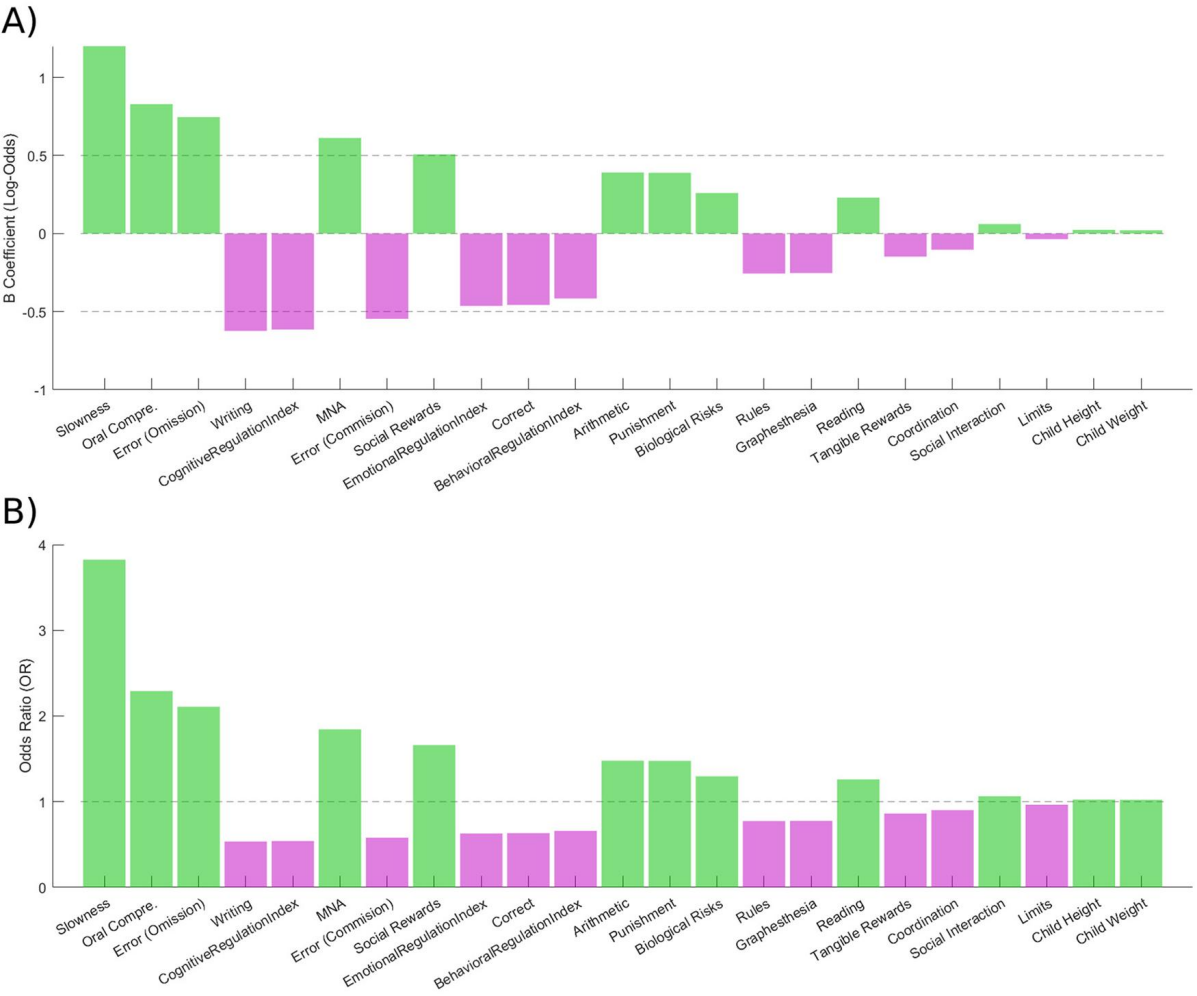
Predictor importance based on logistic regression coefficients (Log-odds). Panel **(A)** shows predictor importance based on logistic regression coefficients (B, log-odds). Positive coefficients indicate a higher likelihood of belonging to the SRB communities, whereas negative coefficients reflect a protective or inverse association. Panel **(B)** displays the corresponding odds ratios (OR), representing the multiplicative change in the odds of SRB exposure for each unit increase in the predictor. Bars depict the strength and direction of each variable’s contribution to group classification. Predictors shown on the *x*-axis correspond to all variables entered simultaneously in the model, including anthropometric, nutritional, neuropsychological, and behavioral measures.

Figure 5A displays the absolute *B* coefficients (log-odds) for all predictors included in the model. Positive coefficients indicate a higher likelihood of belonging to the SRB communities, whereas negative coefficients suggest a protective or inverse association. To facilitate interpretation, the corresponding odds ratios (OR) were also computed (Figure 5B), representing the multiplicative change in the odds of exposure for each unit increase in the predictor. Among the most influential variables were Slowness (*B* = 1.34, OR = 3.82), Oral Comprehension (*B* = 0.83, OR = 2.29), and Omission Errors (*B* = 0.75, OR = 2.12), all positively associated with exposure. In contrast, Writing (*B* = −0.62, OR = 0.54), the Cognitive Regulation Index (*B* = −0.61, OR = 0.54), and Commission Errors (*B* = −0.54, OR = 0.58) were inversely associated. The logistic regression model demonstrated strong classification performance, achieving an overall accuracy of 94% in stratified cross-validation (precision = 0.95, recall = 0.94, F1-score = 0.94).

## Discussion

The present study examined the neurodevelopmental outcomes of children residing in communities surrounding the Sonora River Basin, a region impacted by mining-related contamination, particularly following the 2014 toxic spill. Our findings support the hypothesis that children living in environmentally exposed areas exhibit greater difficulties in executive function and learning domains compared to peers from a non-exposed control group.

Although no significant differences in age, weight, or height were observed across communities, substantial disparities emerged in psychological and neuropsychological outcomes. Children from the Sonora River communities exhibited significantly poorer performance in executive functioning, particularly in the domains of behavioral, emotional, and cognitive regulation, as measured by the BRIEF. These results align with previous research linking chronic exposure to heavy metals, particularly As, Pb, and Mn, to impairments in executive control and self-regulatory processes (7–10, 13).

Children from exposed communities also performed significantly worse on attention tasks from the BANETA, with higher omission errors and lower correct responses, particularly in Cananea, Banámichi, and Baviácora, communities closest to the Buenavista del Cobre mine. This pattern supports the hypothesis of a proximity gradient in neurodevelopmental risk, and parallels findings from other contaminated mining regions in Mexico (10, 13–15, 21, 22, 24). Interestingly, Álamos, the non-exposed control, performed significantly better in several BANETA domains, including attention, writing, and motor slowness, reinforcing the role of environmental exposure in shaping neurocognitive outcomes.

Additionally, emotional and cognitive regulation difficulties were consistently associated with lower academic skills in reading, writing, and arithmetic, as shown by significant correlations between BRIEF indices and BANETA subtests. This aligns with the literature on executive dysfunction mediating academic underachievement in children exposed to neurotoxicants (7–10, 13, 24).

Nutritional status and biological risk factors also emerged as significant contributors. MNA scores, though only marginally different between communities, were positively associated with executive function difficulties, suggesting that even mild nutritional vulnerability may exacerbate cognitive regulation problems. Similarly, biological risk factors during pregnancy and the perinatal period, measured via EDI-based screening, were positively correlated with deficits in executive functioning, highlighting the compounded impact of environmental and prenatal stressors on neurodevelopment (33, 41).

Parenting practices were also found to influence cognitive outcomes in children. Higher scores in punitive practices were associated with greater executive functioning difficulties, while positive parenting behaviors, such as social interaction and the establishment of limits, were positively correlated with academic skills. These findings are consistent with broader research identifying parenting as a critical moderating factor in neurocognitive resilience (31, 33, 41).

The logistic regression model provided valuable insight into the relative contribution of neuropsychological, nutritional, and contextual variables in distinguishing children from environmentally exposed communities. By including a wide range of predictors such as cognitive performance (e.g., writing, attention errors, motor slowness), executive function indices (BRIEF), parenting practices, nutritional status (MNA), and biological risk factors (EDI). The model identified key variables most strongly associated with belonging to the SRB exposure group. Positive B coefficients, such as those for motor slowness (*B* = 1.34), oral comprehension (*B* = 0.83), and omission errors (*B* = 0.75), indicated a higher likelihood of exposure-related classification. In contrast, negative coefficients such as writing ability (*B* = −0.62), the Cognitive Regulation Index (*B* = −0.61), and commission errors (*B* = −0.54) were associated with the non-exposed control group. Importantly, the model demonstrated excellent classification performance (accuracy = 94%, precision = 0.95, recall = 0.94, F1-score = 0.94) in stratified cross-validation. These results suggest that a multifactorial neurodevelopmental profile rather than a single domain characterizes children from contaminated environments. While the cross-sectional nature of the study limits causal inference, the regression analysis advances our understanding of the relative predictive weight of individual variables, laying the groundwork for more targeted interventions. Future research should build on this modeling approach by incorporating longitudinal designs and biological markers to explore how specific risk factors interact over time to shape developmental outcomes.

In light of these findings, it is important to consider not only the caregiving behaviors themselves but also the contextual factors that shape them. Emerging evidence suggests that environmental pollution may significantly affect mental health outcomes, even among adults. Exposure to pollutants such as fine particulate matter (PM_2.5_), Nitrogen dioxide (NO_2_), and sulfur dioxide (SO_2_) has been linked to increased symptoms of anxiety, depression, and schizophrenia, likely through mechanisms involving oxidative stress, systemic inflammation, blood–brain barrier disruption, and epigenetic dysregulation. Light and noise pollution have also been associated with heightened risk of neurodegenerative diseases such as Alzheimer's (42).

Prenatal maternal stress (PNMS) has emerged as a critical determinant of neurodevelopmental outcomes in early childhood. A growing body of research has linked stressors such as natural disasters, maternal depression, and prenatal anxiety to alterations in fetal brain development, particularly in neural systems governing executive function, emotional regulation, and motor control (7, 43). These effects are believed to occur through multiple biological pathways, including dysregulation of the hypothalamic-pituitary-adrenal (HPA) axis, changes in placental functioning, heightened immune activation, and disruptions in the fetal gut microbiome (31). Of particular interest is the concept of maternal mood entropy, marked by fluctuating emotional states which has been identified as a significant predictor of poorer cognitive and language development in children (41). These disruptions often present in early childhood as increased anxiety, attentional deficits, and developmental delays, especially when stress exposure occurs during sensitive gestational windows (8). Considering this multifactorial framework, future studies should investigate how chronic environmental exposures, such as toxic metals interact with caregiver mental health and cognitive functioning. This is particularly salient in socioeconomically disadvantaged and environmentally burdened communities, where cumulative stressors may compromise both caregiver well-being and parenting capacity. Evaluating caregivers' cognitive and emotional health could offer deeper insight into parenting practices and their downstream influence on children's neurodevelopment.

Although punitive parenting practices were similarly reported across all communities, including the control group of Álamos, their relationship with executive functioning in children warrants closer examination. Our findings indicated a consistent association between higher scores in punitive practices and greater executive functioning difficulties, suggesting that even when prevalence is uniform, the psychological impact may vary in relation to other contextual factors, such as stress exposure and regulatory vulnerabilities. Existing literature supports the detrimental impact of punitive discipline on academic outcomes. Studies conducted in school settings have shown that both physical and emotional punishment by parents or teachers are associated with lower academic achievement in children and adolescents (44–46). Taken together, these findings underscore the need to further investigate how discipline styles interact with cognitive regulation capacities, particularly in environmentally and socially vulnerable populations.

Geospatial mapping of BRIEF and BANETA *z*-scores further illustrated the clustering of poorer cognitive outcomes in communities closest to the contaminated zones, visually reinforcing the hypothesized environmental gradient effect. This spatial distribution should be considered in the development of geographically targeted interventions. Beyond the Sonora River region, heavy metal contamination remains a pressing public health issue across Mexico. Studies in other industrial and mining zones, including Torreón, Hidalgo, and Veracruz, have documented elevated levels of lead, arsenic, cadmium, and manganese in environmental matrices and biological samples. These toxicants have been associated with cognitive impairment, behavioral disturbances, and developmental delays in children (14–16). Airborne and dietary exposure routes, particularly through manganese and lead, have also been linked to reduced cognitive performance and adverse reproductive outcomes (18, 19).

Chronic exposure to toxic metals in mining-impacted communities does not operate in isolation, it interacts dynamically with biological and psychosocial vulnerabilities to shape neurodevelopmental trajectories. As highlighted in recent reviews (47, 48), heavy metals such as lead, arsenic, and cadmium induce neurotoxic effects through mechanisms including oxidative stress, neuroinflammation, mitochondrial dysfunction, and disruption of neurotransmitter systems. These effects are particularly detrimental during early childhood, a critical window of rapid brain development. Moreover, biological factors such as malnutrition and micronutrient deficiencies (e.g., iron, zinc, selenium) can exacerbate metal toxicity by increasing absorption and impairing detoxification pathways, further heightening susceptibility to cognitive impairment (48). In parallel, psychosocial stressors, such as poverty, low parental education, inconsistent caregiving, and lack of stimulation contribute to neurodevelopmental risk by dysregulating stress-response systems and limiting protective environmental inputs.

Taken together, the results indicate a multifactorial model of neurodevelopmental risk, wherein environmental exposure interacts with biological vulnerability, nutrition, and caregiving context to shape cognitive and academic outcomes. The pattern of findings highlights the need for interdisciplinary public health strategies that combine environmental remediation with educational, nutritional, and psychosocial support services.

### Limitations and future directions

While this study provides compelling evidence linking environmental exposure to adverse neurodevelopmental outcomes, several limitations must be acknowledged. First, the cross-sectional design restricts our ability to infer causality. Although significant associations were identified between environmental context and cognitive functioning, longitudinal data are required to establish temporal precedence and developmental trajectories over time.

Second, although we included a well-matched control group and controlled for contextual variables such as nutrition, parenting practices, and biological risk, potential confounding variables may remain unmeasured. These may include genetic predispositions, prenatal substance exposures, household income, parental education, or other psychosocial stressors not fully captured by our instruments. Future studies should incorporate more comprehensive demographic and psychosocial assessments to better isolate the effects of environmental contaminants.

A major limitation of the current study is the absence of direct biological exposure assessments. We did not measure individual concentrations of heavy metals (e.g., lead, arsenic, mercury, or manganese) in biological samples such as blood, urine, or hair. This lack of biomarker data limits our ability to quantify exposure levels, assess dose–response relationships, and account for individual variability in toxicokinetics. Additionally, the study did not consider the timing of exposure, which is crucial given the heightened vulnerability of the developing brain during specific sensitive periods. The co-occurrence of multiple metal exposures also raises concerns about synergistic neurotoxic effects, which could not be explored in the current design.

Future studies should adopt an integrated approach that combines biomarker-based assessments with neuropsychological testing and contextual variables. This multidimensional framework would provide stronger evidence for the impact of environmental toxicants and inform evidence-based policy decisions. Furthermore, the results of this study underscore the importance of translating scientific findings into educational policy and school-based interventions. The observed impairments in reading, writing, arithmetic, and attention among children from exposed communities suggest the urgent need to adapt school curricula and incorporate targeted cognitive training within classroom settings. Programs should be developed to strengthen executive function and core academic skills, especially in communities at higher risk due to environmental and contextual vulnerabilities. Schools can serve as a strategic point of intervention by integrating specialized learning supports, teacher training, and regular developmental monitoring.

Emerging evidence supports the effectiveness of multifaceted interventions in mitigating the neurodevelopmental consequences of heavy metal exposure, particularly among children in vulnerable communities. Fatima et al. (47) emphasizes that a combination of medical, nutritional, and behavioral strategies can help counteract the neurotoxic effects of chronic exposure to metals such as lead, arsenic, and cadmium. Interventions focused on antioxidant-rich nutrition, parenting support, and early developmental stimulation have shown promise in reducing cognitive deficits and behavioral dysregulation associated with metal-induced oxidative stress and neuroinflammation. Complementing these findings, Udom et al. (48) highlight that public health responses must be integrative and context-specific, combining environmental remediation, community health education, and developmental surveillance. Their systematic review underscores the need for intersectoral collaboration, linking environmental health, pediatric care, and education systems to reduce long-term harm and promote resilience in exposed populations. These insights are particularly relevant to our findings in the Sonora River Basin, where children's neurodevelopment appears to be shaped not only by environmental toxicants but also by interacting social and nutritional risk factors.

Finally, this study provides evidence that children living in communities surrounding the Sonora River Basin experience significantly greater difficulties in executive functioning and core academic skills compared to children from a non-exposed control group. Specifically, deficits were observed in behavioral, emotional, and cognitive regulation, as well as in attention, writing, and motor processing, with the most pronounced impairments concentrated in communities located nearest to the mining zone. This study underscores the urgent need for integrated public health interventions that address both the neurotoxic effects of heavy metal exposure and the broader structural determinants of health, such as poverty, nutritional vulnerability, and limited access to services. It further emphasizes the need to ensure transparency and public accessibility of information on contamination in the SRB in to strengthen community efforts toward environmental justice (49). Geographic proximity to environmental contamination should be recognized as a key social determinant of child development, guiding equitable allocation of remediation resources, developmental screening programs, and environmental justice policies.

## Data Availability

The raw data supporting the conclusions of this article will be made available by the authors, without undue reservation.

## References

[1] Secretaría de Medio Ambiente y Recursos Naturales (SEMARNAT) (2023). Dictamen diagnóstico ambiental. Secretaría de Medio Ambiente y Recursos Naturales. Available online at: https://www.gob.mx/cms/uploads/attachment/file/859786/Ri_o_Sonora_28_07_23_.pdf (Accessed October 15, 2024).

[2] Diario Oficial de la Federación (DOF) (2022). NORMA Oficial Mexicana NOM-127-SSA1-2021, Agua para uso y consumo humano. Límites permisibles de la calidad del agua. Diario Oficial de la Federación (DOF). Available online at: https://www.dof.gob.mx/nota_detalle.php?codigo=5650705&fecha=02/05/2022 (Accessed October 15, 2024).

[3] Gutierrez-Ruiz M Muro-Puente A Ceniceros-Gómez AE Amaro-Ramírez D Pérez-Manzanera L Martínez-Jardines LG Acid spill impact on Sonora River basin. Part I. Sediments: affected area, pollutant geochemistry and health aspects. J Environ Manag. (2022) 314:115032. 10.1016/j.jenvman.2022.115032 35417836

[4] Mendoza-Lagunas JL Meza-Figueroa DM Martínez-Cinco MA O’Rourke MK Centeno-García E Romero FM Health risk assessment in children by arsenic and mercury pollution of groundwater in a mining area in Sonora, Mexico. J Geosci Environ Prot. (2019) 7(06):90. 10.4236/gep.2019.76008

[5] Romo-Morales D Moreno-Rodríguez V Molina-Freaner F Valencia-Moreno M Ruiz J Minjárez-Osorio C Assessment of geogenic and anthropogenic pollution sources using an aquatic plant along the Sonora river basin: insights from elemental concentrations and Pb isotope signatures. Nat Resour Res. (2020) 29:2773–86. 10.1007/s11053-020-09620-8

[6] Centro Nacional de Prevención y Control de Enfermedades (CENAPRECE). Informe Técnico: Abordaje Toxicológico en Salud Humana. Plan de Justicia Para Cananea-Río sonora. Cananea City: Secretaria de Salud de México (2022).

[7] Collins JM Keane JM Deady C Khashan AS McCarthy FP O’Keeffe GW Prenatal stress impacts foetal neurodevelopment: temporal windows of gestational vulnerability. Neurosci Biobehav Rev. (2024) 164:105793. 10.1016/j.neubiorev.2024.105793 38971516

[8] Dickerson AS Frndak S DeSantiago M Mohan A Smith GS. Environmental exposure disparities and neurodevelopmental risk: a review. Curr Environ Health Rep. (2023) 10(2):73–83. 10.1007/s40572-023-00396-6 37002432 PMC11108231

[9] García-Rico L Meza-Figueroa D Jay Gandolfi A Del Rivero CI Martínez-Cinco MA Meza-Montenegro MM. Health risk assessment and urinary excretion of children exposed to arsenic through drinking water and soils in Sonora, Mexico. Biol Trace Elem Res. (2019) 187:9–21. 10.1007/s12011-018-1347-5 29721859

[10] Briseño-Bugarín J Araujo-Padilla X Escot-Espinoza VM Cardoso-Ortiz J Flores de la Torre JA & López-Luna A. Lead (pb) pollution in soil: a systematic review and meta-analysis of contamination grade and health risk in Mexico. Environments. (2024) 11(3):43. 10.3390/environments11030043

[11] Gu Q Liu J Zhang X Huang A Yu X Wu K Association between heavy metals exposure and risk of attention deficit hyperactivity disorder (ADHD) in children: a systematic review and meta-analysis. Eur Child Adolesc Psychiatry. (2025) 34:1–21. 10.1007/s00787-024-02546-z 39126497

[12] Notario-Barandiaran L Díaz-Coto S Jimenez-Redondo N Guxens M Vrijheid M Andiarena A Latent childhood exposure to mixtures of metals and neurodevelopmental outcomes in 4–5-year-old children living in Spain. Exp Health. (2024) 16(4):1053–66. 10.1007/s12403-023-00610-8

[13] Riojas-Rodríguez H Solís-Vivanco R Schilmann A Montes S Rodríguez S Ríos C Intellectual function in Mexican children living in a mining area and environmentally exposed to manganese. Environ Health Perspect. (2010) 118(10):1465–70. 10.1289/ehp.0901229 20936744 PMC2957930

[14] Rodríguez-Agudelo Y Riojas-Rodríguez H Ríos C Rosas I Pedraza ES Miranda J Motor alterations associated with exposure to manganese in the environment in Mexico. Sci Total Environ. (2006) 368(2-3):542–56. 10.1016/j.scitotenv.2006.03.025 16793118

[15] Roy A Kordas K Lopez P Rosado JL Cebrian ME Vargas GG Association between arsenic exposure and behavior among first-graders from Torreon, Mexico. Environ Res. (2011) 111(5):670–6. 10.1016/j.envres.2011.03.003 21439564

[16] Santos-Burgoa C Rios C Mercado LA Arechiga-Serrano R Cano-Valle F Eden-Wynter RA Exposure to manganese: health effects on the general population, a pilot study in central Mexico. Environ Res. (2001) 85(2):90–104. 10.1006/enrs.2000.4108 11161659

[17] Trejo-Acevedo A Díaz-Barriga F Carrizales L Domínguez G Costilla R Ize-Lema I Exposure assessment of persistent organic pollutants and metals in Mexican children. Chemosphere. (2009) 74(7):974–80. 10.1016/j.chemosphere.2008.10.030 19091374

[18] Velandia-Aquino LB Botello AV Ponce-Vélez G Namihira-Santillán PE Villanueva-Fragoso S. Vertical distribution of potentially toxic metals and PAHs in the alvarado lagoon, veracruz in the southern Gulf of Mexico. Estuaries Coast. (2024) 47(8):2589–602. 10.1007/s12237-023-01307-6

[19] Rivera Carvajal R Duarte-Tagles H & Idrovo ÁJ. Mining leachate contamination and subfecundity among women living near the USA–Mexico border. Environ Geochem Health. (2019) 41:2169–78. 10.1007/s10653-019-00275-w(0123456789().,-volV)(0123456789().,-volV) 30868353

[20] Acosta-Saavedra LC Moreno ME Rodríguez-Kessler T Luna A Gomez R Arias-Salvatierra D Environmental exposure to lead and mercury in Mexican children: a real health problem. Toxicol Mech Methods. (2011) 21(9):656–66. 10.3109/15376516.2011.620997 21981766

[21] Calderon J Navarro ME Jimenez-Capdeville ME Santos-Diaz MA Golden A Rodriguez-Leyva I Exposure to arsenic and lead and neuropsychological development in Mexican children. Environ Res. (2001) 85(2):69–76. 10.1006/enrs.2000.4106 11161656

[22] García-Chimalpopoca Z Hernández-Bonilla D Cortez-Lugo M Escamilla-Núñez C Schilmann A Riojas-Rodríguez H Verbal memory and learning in schoolchildren exposed to manganese in Mexico. Neurotox Res. (2019) 36:827–35. 10.1007/s12640-019-00037-7 31148117

[23] Torres-Agustín R Rodríguez-Agudelo Y Schilmann A Solís-Vivanco R Montes S Riojas-Rodríguez H Effect of environmental manganese exposure on verbal learning and memory in Mexican children. Environ Res. (2013) 121:39–44. 10.1016/j.envres.2012.10.007 23141434

[24] Hicks SD Wang M Fry K Doraiswamy V Wohlford EM. Neurodevelopmental delay diagnosis rates are increased in a region with aerial pesticide application. Front Pediatr. (2017) 5:116. 10.3389/fped.2017.00116 28596952 PMC5443159

[25] Bauer JA Romano ME Jackson BP Bellinger D Korrick S Karagas MR. Associations of perinatal metal and metalloid exposures with early child behavioral development over time in the New Hampshire birth cohort study. Exp Health. (2024) 16(1):135–48. 10.1007/s12403-023-00543-2 PMC11060719 38694196

[26] Dórea JG. Environmental exposure to low-level lead (pb) co-occurring with other neurotoxicants in early life and neurodevelopment of children. Environ Res. (2019) 177:108641. 10.1016/j.envres.2019.108641 31421445

[27] Bora BK Ramos-Crawford AL Sikorskii A Boivin MJ Lez DM Mumba-Ngoyi D Concurrent exposure to heavy metals and cognition in school-age children in Congo-Kinshasa: a complex overdue research agenda. Brain Res Bull. (2019) 145:81–6. 10.1016/j.brainresbull.2018.06.013 29944947 PMC6631038

[28] Soetrisno FN Delgado-Saborit JM. Chronic exposure to heavy metals from informal e-waste recycling plants and children’s attention, executive function and academic performance. Sci Total Environ. (2020) 717:137099. 10.1016/j.scitotenv.2020.137099 32092800

[29] Symanski E Whitworth KW Han I Rammah A Alvarez J Moussa I Assessing metal exposures among children living in environmental justice communities near metal recycling facilities in Houston, Texas. Environ Just. (2024) 17(2):128–42. 10.1089/env.2022.0023 PMC12302942 40726841

[30] Silveira PP More L Gottfried C. Editorial: gene and environment interactions in neurodevelopmental disorders. Front Behav Neurosci (2022) 16:893662. 10.3389/fnbeh.2022.893662 35431834 PMC9008214

[31] Ünsel-Bolat G Yıldırım S Kılıçaslan F Caparros-Gonzalez RA. Natural disasters as a maternal prenatal stressor and children’s neurodevelopment: a systematic review. Behav Sci. (2024) 14(11):1054. 10.3390/bs14111054 39594354 PMC11590888

[32] Yesildemir O Celik MN. Association between pre-and postnatal exposure to endocrine-disrupting chemicals and birth and neurodevelopmental outcomes: an extensive review. Clin Exp Pediatr. (2024) 67(7):328. 10.3345/cep.2023.00941 37986566 PMC11222910

[33] Farías P Hernández-Bonilla D Moreno-Macías H Montes-López S Schnaas L Texcalac-Sangrador JL Prenatal co-exposure to manganese, mercury, and lead, and neurodevelopment in children during the first year of life. Int J Environ Res Public Health. (2022) 19(20):13020. 10.3390/ijerph192013020 36293596 PMC9603303

[34] Yáñez TMG Prieto CDMB. Batería Neuropsicológica Para la Evaluación de los Trastornos del Aprendizaje [Neuropsychological Battery for Assessment of Learning Disabilities]. México: Manual Moderno (2013).

[35] Gioia GA Isquith PK Retzlaff PD Espy KA. Confirmatory factor analysis of the behavior rating inventory of executive function (BRIEF) in a clinical sample. Child Neuropsychol. (2002) 8(4):249–57. 10.1076/chin.8.4.249.13513 12759822

[36] García-Anacleto A Salvador-Cruz J. Adaptación y validación en población mexicana de la escala BRIEF-P. Cuadernos Neuropsicol/Panamerican J Neuropsychol. (2017) 11(3):42–60. 10.7714/CNPS/11.3.202

[37] López CF. Inventario de prácticas de crianza. In: Morales ECS Martínez RMJ, (Dirs.) Prevención de las Conductas Adictivas a Través de la Atención del Comportamiento Infantil Para la Crianza Positiva. Manual del Terapeuta. México: CENADIC-SSA (2013). p. 14–9.

[38] Grot M Białek-Dratwa A Krupa-Kotara K Grajek M Nigowski M Szczepańska E Feeding problems, eating disorders, and nutritional status of Polish children and adolescents with neurodevelopmental disorders–a cross-sectional pilot study. Pediatr Pol-Pol J Paediatr. (2024) 99(1):37–45. 10.5114/polp.2024.135986

[39] Salach N Brunson C Zhang A Sgambat K. Investigation of normalized protein catabolic rate as a marker of nutritional status in infants and children receiving chronic hemodialysis: a longitudinal cohort study. Pediatr Nephrol. (2025) 40:3485–3493. 10.1007/s00467-025-06859-2 40549190

[40] Rizzoli-Córdoba A Schnaas-Arrieta L Liendo-Vallejos S Buenrostro-Márquez G Romo-Pardo B Carreón-García J Validación de un instrumento para la detección oportuna de problemas de desarrollo en menores de 5 años en México. Bol Méd Hosp Infant Méx. (2013) 70(3):195–208. Available online at: https://www.medigraphic.com/cgi-bin/new/resumen.cgi?IDARTICULO=42432

[41] Howland MA Sandman CA Davis EP Stern HS Phelan M Baram TZ Prenatal maternal mood entropy is associated with child neurodevelopment. Emotion. (2021) 21(3):489. 10.1037/emo0000726 32202848 PMC10491461

[42] Tota M Karska J Kowalski S Piatek N Pszczołowska M Mazur K Environmental pollution and extreme weather conditions: insights into the effect on mental health. Front Psychiatry. (2024) 15:1389051. 10.3389/fpsyt.2024.1389051 38863619 PMC11165707

[43] Nomura Y Rompala G Pritchett L Aushev V Chen J Hurd YL. Natural disaster stress during pregnancy is linked to reprogramming of the placenta transcriptome in relation to anxiety and stress hormones in young offspring. Mol Psychiatry. (2021) 26(11):6520–30. 10.1038/s41380-021-01123-z 33981007 PMC8586067

[44] Ahmad M Khalid MN Zafar T. Investigating the relationship between parental punishment and academic achievement: a case study of secondary level students in Sargodha, Pakistan. UMT Educ Rev. (2022) 5(2):86–101. 10.32350/uer.52.05

[45] Anwar S Zeeshan A Gill SA Raza SA Naqvi SAH. Impact of corporal punishment on students’ academic performance at elementary schools level. Psychol Educ. (2021) 58(3):1846–52. 10.17762/pae.v58i3.3970

[46] Hwang S Allen JL Kokosi T Bird E. To what extent does punishment insensitivity explain the relationship between callous-unemotional traits and academic performance in secondary school students? Br J Educ Psychol. (2021) 91(3):811–26. 10.1111/bjep.12394 33270221

[47] Fatima G Raza AM Dhole P. Heavy metal exposure and its health implications: a comprehensive review. Indian J Clin Biochem. (2025):1–29. 10.1007/s12291-025-01322-339835227

[48] Udom GJ Iyaye D Oritsemuelebi B Nwanaforo E Bede-Ojimadu O Abdulai PM Public health concerns of multifaceted exposures to metal and metalloid mixtures: a systematic review. Environ Monit Assess. (2025) 197(5):502. 10.1007/s10661-025-13963-1 40172706

[49] Robles-Morua A Avila-Chauvet L Díaz-Caravantes RE Vizuete-Jaramillo E Lutz-Ley A Duarte-Tagles H The socio-environmental observatory: an information platform for transparency, participatory governance, and environmental justice in the Sonora River Basin, Mexico. Socio-Ecol Pract Res. (2025) 7:1–17. 10.1007/s42532-025-00226-z

[50] LeraL SánchezH ÁngelB AlbalaC. Mini nutritional assessment short-form: validation in five Latin American cities. SABE study. J Nutr Health Aging. (2016) 20(8):797–805. 10.1007/s12603-016-0696-z27709228

