# Supplementary material for: Neurodevelopmental impact of mining-related contamination in children from the Sonora river basin
Source: Front Pediatr. 2025 Dec 5;13:1681071. doi: 10.3389/fped.2025.1681071 (PMC12714966; doi:10.3389/fped.2025.1681071)
Supplement: Supplementary file 1 [file Datasheet1.pdf]

Figure 1

Bonferroni post-hoc comparisons of Age (years) among communities.

Post Hoc Comparisons - Community ▼

		Mean Difference	SE	t	p_{bonf}
Aconchi	Alamos	−0.126	0.481	−0.262	1.000
	Banamichi	−0.072	0.445	−0.162	1.000
	Baviacora	0.377	0.428	0.882	1.000
	Cananea	0.169	0.445	0.380	1.000
	Huepac	1.301	0.526	2.476	0.392
	San Felipe	−0.213	0.481	−0.443	1.000
	Ures	0.748	0.436	1.714	1.000
Alamos	Banamichi	0.054	0.537	0.101	1.000
	Baviacora	0.503	0.522	0.964	1.000
	Cananea	0.295	0.537	0.550	1.000
	Huepac	1.428	0.605	2.361	0.534
	San Felipe	−0.087	0.567	−0.153	1.000
	Ures	0.874	0.529	1.652	1.000
Banamichi	Baviacora	0.449	0.489	0.919	1.000
	Cananea	0.241	0.505	0.478	1.000
	Huepac	1.374	0.577	2.382	0.505
	San Felipe	−0.141	0.537	−0.263	1.000
	Ures	0.820	0.496	1.651	1.000
Baviacora	Cananea	−0.208	0.489	−0.425	1.000
	Huepac	0.924	0.563	1.641	1.000
	San Felipe	−0.590	0.522	−1.131	1.000
	Ures	0.370	0.481	0.771	1.000
Cananea	Huepac	1.132	0.577	1.963	1.000
	San Felipe	−0.382	0.537	−0.713	1.000
	Ures	0.578	0.496	1.165	1.000
Huepac	San Felipe	−1.514	0.605	−2.504	0.363
	Ures	−0.554	0.569	−0.972	1.000
San Felipe	Ures	0.961	0.529	1.817	1.000

Note. P-value adjusted for comparing a family of 8

Figure 2

Bonferroni post-hoc comparisons of Weight (kg) among communities.

Post Hoc Comparisons - Community ▼

		Mean Difference	SE	t	p_{bonf}
Aconchi	Alamos	0.713	3.809	0.187	1.000
	Banamichi	−3.193	4.246	−0.752	1.000
	Baviacora	−5.526	3.684	−1.500	1.000
	Cananea	−1.717	3.684	−0.466	1.000
	Huepac	5.126	3.958	1.295	1.000
	San Felipe	−5.050	3.881	−1.301	1.000
	Ures	−4.675	4.044	−1.156	1.000
Alamos	Banamichi	−3.906	4.816	−0.811	1.000
	Baviacora	−6.239	4.329	−1.441	1.000
	Cananea	−2.430	4.329	−0.561	1.000
	Huepac	4.413	4.565	0.967	1.000
	San Felipe	−5.763	4.497	−1.282	1.000
	Ures	−5.388	4.639	−1.161	1.000
Banamichi	Baviacora	−2.333	4.717	−0.495	1.000
	Cananea	1.476	4.717	0.313	1.000
	Huepac	8.319	4.934	1.686	1.000
	San Felipe	−1.857	4.872	−0.381	1.000
	Ures	−1.482	5.004	−0.296	1.000
Baviacora	Cananea	3.810	4.219	0.903	1.000
	Huepac	10.653	4.461	2.388	0.507
	San Felipe	0.476	4.392	0.108	1.000
	Ures	0.851	4.537	0.188	1.000
Cananea	Huepac	6.843	4.461	1.534	1.000
	San Felipe	−3.333	4.392	−0.759	1.000
	Ures	−2.958	4.537	−0.652	1.000
Huepac	San Felipe	−10.176	4.624	−2.201	0.818
	Ures	−9.801	4.762	−2.058	1.000
San Felipe	Ures	0.375	4.698	0.080	1.000

Note. P-value adjusted for comparing a family of 8

Figure 3

Bonferroni post-hoc comparisons of Height (cm) among communities.

Post Hoc Comparisons - Community

		Mean Difference	SE	t	p_{bonf}
Aconchi	Alamos	−3.953	3.969	−0.996	1.000
	Banamichi	−3.019	4.764	−0.634	1.000
	Baviacora	−4.388	3.906	−1.123	1.000
	Cananea	−3.960	4.496	−0.881	1.000
	Huepac	3.603	4.194	0.859	1.000
	San Felipe	−1.044	4.194	−0.249	1.000
	Ures	0.192	4.194	0.046	1.000
Alamos	Banamichi	0.933	5.270	0.177	1.000
	Baviacora	−0.436	4.509	−0.097	1.000
	Cananea	−0.007	5.029	−0.001	1.000
	Huepac	7.556	4.761	1.587	1.000
	San Felipe	2.909	4.761	0.611	1.000
	Ures	4.144	4.761	0.870	1.000
Banamichi	Baviacora	−1.369	5.222	−0.262	1.000
	Cananea	−0.940	5.677	−0.166	1.000
	Huepac	6.623	5.441	1.217	1.000
	San Felipe	1.975	5.441	0.363	1.000
	Ures	3.211	5.441	0.590	1.000
Baviacora	Cananea	0.429	4.979	0.086	1.000
	Huepac	7.992	4.708	1.697	1.000
	San Felipe	3.345	4.708	0.710	1.000
	Ures	4.580	4.708	0.973	1.000
Cananea	Huepac	7.563	5.208	1.452	1.000
	San Felipe	2.916	5.208	0.560	1.000
	Ures	4.151	5.208	0.797	1.000
Huepac	San Felipe	−4.647	4.950	−0.939	1.000
	Ures	−3.412	4.950	−0.689	1.000
San Felipe	Ures	1.235	4.950	0.250	1.000

Note. P-value adjusted for comparing a family of 8

Figure 4

Dunn's post-hoc comparisons of MNA (Mini Nutritional Assessment) among communities.

Dunn's Post Hoc Comparisons - Community ▼

Comparison	z	W_i	W_j	p	p_{bonf}	p_{holm}
Aconchi - Alamos	−0.183	108.712	111.717	0.855	1.000	1.000
Aconchi - Banamichi	−2.331	108.712	144.207	0.020*	0.553	0.415
Aconchi - Baviacora	−0.730	108.712	119.379	0.466	1.000	1.000
Aconchi - Cananea	−3.183	108.712	157.172	0.001**	0.041*	0.039*
Aconchi - Huepac	0.881	108.712	92.889	0.379	1.000	1.000
Aconchi - San Felipe	0.580	108.712	99.174	0.562	1.000	1.000
Aconchi - Ures	−0.467	108.712	115.677	0.640	1.000	1.000
Alamos - Banamichi	−1.771	111.717	144.207	0.077	1.000	1.000
Alamos - Baviacora	−0.429	111.717	119.379	0.668	1.000	1.000
Alamos - Cananea	−2.478	111.717	157.172	0.013*	0.370	0.317
Alamos - Huepac	0.911	111.717	92.889	0.362	1.000	1.000
Alamos - San Felipe	0.647	111.717	99.174	0.517	1.000	1.000
Alamos - Ures	−0.219	111.717	115.677	0.827	1.000	1.000
Banamichi - Baviacora	1.485	144.207	119.379	0.138	1.000	1.000
Banamichi - Cananea	−0.751	144.207	157.172	0.452	1.000	1.000
Banamichi - Huepac	2.603	144.207	92.889	0.009**	0.259	0.231
Banamichi - San Felipe	2.455	144.207	99.174	0.014*	0.395	0.324
Banamichi - Ures	1.681	144.207	115.677	0.093	1.000	1.000
Baviacora - Cananea	−2.260	119.379	157.172	0.024*	0.667	0.476
Baviacora - Huepac	1.376	119.379	92.889	0.169	1.000	1.000
Baviacora - San Felipe	1.132	119.379	99.174	0.258	1.000	1.000
Baviacora - Ures	0.225	119.379	115.677	0.822	1.000	1.000
Cananea - Huepac	3.261	157.172	92.889	0.001**	0.031*	0.031*
Cananea - San Felipe	3.162	157.172	99.174	0.002**	0.044*	0.041*
Cananea - Ures	2.445	157.172	115.677	0.014*	0.406	0.324
Huepac - San Felipe	−0.304	92.889	99.174	0.761	1.000	1.000
Huepac - Ures	−1.171	92.889	115.677	0.242	1.000	1.000
San Felipe - Ures	−0.913	99.174	115.677	0.361	1.000	1.000

* p < .05, ** p < .01

Figure 5

Dunn's post-hoc comparisons of Biological Risks among communities.

Dunn's Post Hoc Comparisons - Community ▼

Comparison	z	W_i	W_j	p	p_{bonf}	p_{holm}
Aconchi - Alamos	−1.848	121.202	152.217	0.065	1.000	1.000
Aconchi - Banamichi	0.423	121.202	114.638	0.673	1.000	1.000
Aconchi - Baviacora	0.883	121.202	108.030	0.377	1.000	1.000
Aconchi - Cananea	−0.902	121.202	135.207	0.367	1.000	1.000
Aconchi - Huepac	1.599	121.202	91.889	0.110	1.000	1.000
Aconchi - San Felipe	1.157	121.202	101.783	0.247	1.000	1.000
Aconchi - Ures	−0.159	121.202	123.613	0.874	1.000	1.000
Alamos - Banamichi	2.008	152.217	114.638	0.045*	1.000	1.000
Alamos - Baviacora	2.427	152.217	108.030	0.015*	0.426	0.396
Alamos - Cananea	0.909	152.217	135.207	0.363	1.000	1.000
Alamos - Huepac	2.860	152.217	91.889	0.004**	0.119	0.119
Alamos - San Felipe	2.552	152.217	101.783	0.011*	0.300	0.289
Alamos - Ures	1.551	152.217	123.613	0.121	1.000	1.000
Banamichi - Baviacora	0.387	114.638	108.030	0.699	1.000	1.000
Banamichi - Cananea	−1.169	114.638	135.207	0.243	1.000	1.000
Banamichi - Huepac	1.131	114.638	91.889	0.258	1.000	1.000
Banamichi - San Felipe	0.687	114.638	101.783	0.492	1.000	1.000
Banamichi - Ures	−0.518	114.638	123.613	0.604	1.000	1.000
Baviacora - Cananea	−1.593	108.030	135.207	0.111	1.000	1.000
Baviacora - Huepac	0.822	108.030	91.889	0.411	1.000	1.000
Baviacora - San Felipe	0.343	108.030	101.783	0.731	1.000	1.000
Baviacora - Ures	−0.929	108.030	123.613	0.353	1.000	1.000
Cananea - Huepac	2.154	135.207	91.889	0.031*	0.875	0.781
Cananea - San Felipe	1.786	135.207	101.783	0.074	1.000	1.000
Cananea - Ures	0.670	135.207	123.613	0.503	1.000	1.000
Huepac - San Felipe	−0.469	91.889	101.783	0.639	1.000	1.000
Huepac - Ures	−1.597	91.889	123.613	0.110	1.000	1.000
San Felipe - Ures	−1.183	101.783	123.613	0.237	1.000	1.000

* p < .05, ** p < .01

Figure 6

Dunn’s post-hoc comparisons of Punishment among communities.

Dunn's Post Hoc Comparisons - Community ▼

Comparison	z	W_i	W_j	p	p_{bonf}	p_{holm}
Aconchi - Alamos	−2.066	110.240	145.391	0.039*	1.000	1.000
Aconchi - Banamichi	−0.403	110.240	116.661	0.687	1.000	1.000
Aconchi - Baviacora	0.050	110.240	109.468	0.960	1.000	1.000
Aconchi - Cananea	−0.799	110.240	122.828	0.424	1.000	1.000
Aconchi - Huepac	−0.606	110.240	121.500	0.544	1.000	1.000
Aconchi - San Felipe	0.452	110.240	102.543	0.651	1.000	1.000
Aconchi - Ures	−0.981	110.240	125.355	0.327	1.000	1.000
Alamos - Banamichi	1.503	145.391	116.661	0.133	1.000	1.000
Alamos - Baviacora	1.922	145.391	109.468	0.055	1.000	1.000
Alamos - Cananea	1.190	145.391	122.828	0.234	1.000	1.000
Alamos - Huepac	1.118	145.391	121.500	0.264	1.000	1.000
Alamos - San Felipe	2.139	145.391	102.543	0.032*	0.908	0.908
Alamos - Ures	1.072	145.391	125.355	0.284	1.000	1.000
Banamichi - Baviacora	0.406	116.661	109.468	0.685	1.000	1.000
Banamichi - Cananea	−0.343	116.661	122.828	0.732	1.000	1.000
Banamichi - Huepac	−0.236	116.661	121.500	0.814	1.000	1.000
Banamichi - San Felipe	0.738	116.661	102.543	0.460	1.000	1.000
Banamichi - Ures	−0.491	116.661	125.355	0.623	1.000	1.000
Baviacora - Cananea	−0.761	109.468	122.828	0.446	1.000	1.000
Baviacora - Huepac	−0.598	109.468	121.500	0.550	1.000	1.000
Baviacora - San Felipe	0.370	109.468	102.543	0.711	1.000	1.000
Baviacora - Ures	−0.921	109.468	125.355	0.357	1.000	1.000
Cananea - Huepac	0.065	122.828	121.500	0.948	1.000	1.000
Cananea - San Felipe	1.069	122.828	102.543	0.285	1.000	1.000
Cananea - Ures	−0.144	122.828	125.355	0.885	1.000	1.000
Huepac - San Felipe	0.887	121.500	102.543	0.375	1.000	1.000
Huepac - Ures	−0.191	121.500	125.355	0.848	1.000	1.000
San Felipe - Ures	−1.220	102.543	125.355	0.222	1.000	1.000

* $p < .05$

Figure 7

Dunn’s post-hoc comparisons of Material Rewards among communities.

Dunn's Post Hoc Comparisons - Community

Comparison	z	W_i	W_j	p	p_{bonf}	p_{holm}
Aconchi - Alamos	−0.298	130.837	135.913	0.765	1.000	1.000
Aconchi - Banamichi	−0.843	130.837	144.250	0.399	1.000	1.000
Aconchi - Baviacora	0.479	130.837	123.452	0.632	1.000	1.000
Aconchi - Cananea	2.760	130.837	87.397	0.006**	0.162	0.150
Aconchi - Huepac	1.400	130.837	104.833	0.161	1.000	1.000
Aconchi - San Felipe	0.732	130.837	118.391	0.464	1.000	1.000
Aconchi - Ures	2.650	130.837	90.000	0.008**	0.225	0.201
Alamos - Banamichi	−0.436	135.913	144.250	0.663	1.000	1.000
Alamos - Baviacora	0.667	135.913	123.452	0.505	1.000	1.000
Alamos - Cananea	2.559	135.913	87.397	0.011*	0.294	0.252
Alamos - Huepac	1.454	135.913	104.833	0.146	1.000	1.000
Alamos - San Felipe	0.875	135.913	118.391	0.382	1.000	1.000
Alamos - Ures	2.457	135.913	90.000	0.014*	0.393	0.323
Banamichi - Baviacora	1.175	144.250	123.452	0.240	1.000	1.000
Banamichi - Cananea	3.160	144.250	87.397	0.002**	0.044*	0.044*
Banamichi - Huepac	1.921	144.250	104.833	0.055	1.000	1.000
Banamichi - San Felipe	1.353	144.250	118.391	0.176	1.000	1.000
Banamichi - Ures	3.064	144.250	90.000	0.002**	0.061	0.059
Baviacora - Cananea	2.055	123.452	87.397	0.040*	1.000	0.877
Baviacora - Huepac	0.925	123.452	104.833	0.355	1.000	1.000
Baviacora - San Felipe	0.271	123.452	118.391	0.787	1.000	1.000
Baviacora - Ures	1.939	123.452	90.000	0.052	1.000	1.000
Cananea - Huepac	−0.856	87.397	104.833	0.392	1.000	1.000
Cananea - San Felipe	−1.635	87.397	118.391	0.102	1.000	1.000
Cananea - Ures	−0.148	87.397	90.000	0.882	1.000	1.000
Huepac - San Felipe	−0.634	104.833	118.391	0.526	1.000	1.000
Huepac - Ures	0.737	104.833	90.000	0.461	1.000	1.000
San Felipe - Ures	1.519	118.391	90.000	0.129	1.000	1.000

* p < .05, ** p < .01

Figure 8

Dunn's post-hoc comparisons of Social Interaction among communities.

Dunn's Post Hoc Comparisons - Community ▼

Comparison	z	W_i	W_j	p	p_{bonf}	p_{holm}
Aconchi - Alamos	0.845	122.529	108.239	0.398	1.000	1.000
Aconchi - Banamichi	0.852	122.529	109.036	0.394	1.000	1.000
Aconchi - Baviacora	−0.782	122.529	134.516	0.434	1.000	1.000
Aconchi - Cananea	0.309	122.529	117.690	0.757	1.000	1.000
Aconchi - Huepac	1.200	122.529	100.361	0.230	1.000	1.000
Aconchi - San Felipe	−0.045	122.529	123.283	0.964	1.000	1.000
Aconchi - Ures	0.437	122.529	115.839	0.662	1.000	1.000
Alamos - Banamichi	−0.042	108.239	109.036	0.967	1.000	1.000
Alamos - Baviacora	−1.414	108.239	134.516	0.157	1.000	1.000
Alamos - Cananea	−0.501	108.239	117.690	0.616	1.000	1.000
Alamos - Huepac	0.371	108.239	100.361	0.711	1.000	1.000
Alamos - San Felipe	−0.755	108.239	123.283	0.450	1.000	1.000
Alamos - Ures	−0.409	108.239	115.839	0.683	1.000	1.000
Banamichi - Baviacora	−1.447	109.036	134.516	0.148	1.000	1.000
Banamichi - Cananea	−0.484	109.036	117.690	0.629	1.000	1.000
Banamichi - Huepac	0.425	109.036	100.361	0.671	1.000	1.000
Banamichi - San Felipe	−0.750	109.036	123.283	0.453	1.000	1.000
Banamichi - Ures	−0.386	109.036	115.839	0.699	1.000	1.000
Baviacora - Cananea	0.965	134.516	117.690	0.335	1.000	1.000
Baviacora - Huepac	1.707	134.516	100.361	0.088	1.000	1.000
Baviacora - San Felipe	0.604	134.516	123.283	0.546	1.000	1.000
Baviacora - Ures	1.089	134.516	115.839	0.276	1.000	1.000
Cananea - Huepac	0.855	117.690	100.361	0.392	1.000	1.000
Cananea - San Felipe	−0.297	117.690	123.283	0.767	1.000	1.000
Cananea - Ures	0.106	117.690	115.839	0.916	1.000	1.000
Huepac - San Felipe	−1.079	100.361	123.283	0.281	1.000	1.000
Huepac - Ures	−0.773	100.361	115.839	0.439	1.000	1.000
San Felipe - Ures	0.401	123.283	115.839	0.689	1.000	1.000

Figure 9

Dunn's post-hoc comparisons of Rules among communities.

Dunn's Post Hoc Comparisons - Community ▼

Comparison	z	W_i	W_j	p	p_{bonf}	p_{holm}
Aconchi - Alamos	−0.899	117.115	132.196	0.369	1.000	1.000
Aconchi - Banamichi	−0.661	117.115	127.500	0.508	1.000	1.000
Aconchi - Baviacora	0.575	117.115	108.371	0.565	1.000	1.000
Aconchi - Cananea	0.051	117.115	116.328	0.960	1.000	1.000
Aconchi - Huepac	0.264	117.115	112.278	0.792	1.000	1.000
Aconchi - San Felipe	1.296	117.115	95.370	0.195	1.000	1.000
Aconchi - Ures	−0.958	117.115	131.677	0.338	1.000	1.000
Alamos - Banamichi	0.249	132.196	127.500	0.803	1.000	1.000
Alamos - Baviacora	1.292	132.196	108.371	0.196	1.000	1.000
Alamos - Cananea	0.848	132.196	116.328	0.396	1.000	1.000
Alamos - Huepac	0.945	132.196	112.278	0.345	1.000	1.000
Alamos - San Felipe	1.864	132.196	95.370	0.062	1.000	1.000
Alamos - Ures	0.028	132.196	131.677	0.978	1.000	1.000
Banamichi - Baviacora	1.095	127.500	108.371	0.273	1.000	1.000
Banamichi - Cananea	0.629	127.500	116.328	0.529	1.000	1.000
Banamichi - Huepac	0.752	127.500	112.278	0.452	1.000	1.000
Banamichi - San Felipe	1.704	127.500	95.370	0.088	1.000	1.000
Banamichi - Ures	−0.239	127.500	131.677	0.811	1.000	1.000
Baviacora - Cananea	−0.460	108.371	116.328	0.646	1.000	1.000
Baviacora - Huepac	−0.197	108.371	112.278	0.844	1.000	1.000
Baviacora - San Felipe	0.705	108.371	95.370	0.481	1.000	1.000
Baviacora - Ures	−1.369	108.371	131.677	0.171	1.000	1.000
Cananea - Huepac	0.201	116.328	112.278	0.840	1.000	1.000
Cananea - San Felipe	1.120	116.328	95.370	0.263	1.000	1.000
Cananea - Ures	−0.887	116.328	131.677	0.375	1.000	1.000
Huepac - San Felipe	0.802	112.278	95.370	0.423	1.000	1.000
Huepac - Ures	−0.977	112.278	131.677	0.329	1.000	1.000
San Felipe - Ures	−1.969	95.370	131.677	0.049*	1.000	1.000

* $p < .05$

Figure 10

Dunn's post-hoc comparisons of Social Rewards among communities.

Dunn's Post Hoc Comparisons - Community ▼

Comparison	z	W_i	W_j	p	p_{bonf}	p_{holm}
Aconchi - Alamos	−0.175	112.029	114.913	0.861	1.000	1.000
Aconchi - Banamichi	−1.287	112.029	132.111	0.198	1.000	1.000
Aconchi - Baviacora	−0.127	112.029	113.919	0.899	1.000	1.000
Aconchi - Cananea	0.182	112.029	109.259	0.856	1.000	1.000
Aconchi - Huepac	−0.015	112.029	112.306	0.988	1.000	1.000
Aconchi - San Felipe	−0.278	112.029	116.609	0.781	1.000	1.000
Aconchi - Ures	−1.260	112.029	130.839	0.208	1.000	1.000
Alamos - Banamichi	−0.921	114.913	132.111	0.357	1.000	1.000
Alamos - Baviacora	0.055	114.913	113.919	0.956	1.000	1.000
Alamos - Cananea	0.308	114.913	109.259	0.758	1.000	1.000
Alamos - Huepac	0.126	114.913	112.306	0.900	1.000	1.000
Alamos - San Felipe	−0.087	114.913	116.609	0.930	1.000	1.000
Alamos - Ures	−0.880	114.913	130.839	0.379	1.000	1.000
Banamichi - Baviacora	1.051	132.111	113.919	0.293	1.000	1.000
Banamichi - Cananea	1.299	132.111	109.259	0.194	1.000	1.000
Banamichi - Huepac	0.990	132.111	112.306	0.322	1.000	1.000
Banamichi - San Felipe	0.831	132.111	116.609	0.406	1.000	1.000
Banamichi - Ures	0.073	132.111	130.839	0.941	1.000	1.000
Baviacora - Cananea	0.274	113.919	109.259	0.784	1.000	1.000
Baviacora - Huepac	0.083	113.919	112.306	0.934	1.000	1.000
Baviacora - San Felipe	−0.149	113.919	116.609	0.882	1.000	1.000
Baviacora - Ures	−1.013	113.919	130.839	0.311	1.000	1.000
Cananea - Huepac	−0.154	109.259	112.306	0.877	1.000	1.000
Cananea - San Felipe	−0.400	109.259	116.609	0.689	1.000	1.000
Cananea - Ures	−1.270	109.259	130.839	0.204	1.000	1.000
Huepac - San Felipe	−0.208	112.306	116.609	0.835	1.000	1.000
Huepac - Ures	−0.951	112.306	130.839	0.342	1.000	1.000
San Felipe - Ures	−0.786	116.609	130.839	0.432	1.000	1.000

Figure 11

Dunn's post-hoc comparisons of Limits among communities.

Dunn's Post Hoc Comparisons - Community

Comparison	z	W_i	W_j	p	p_{bonf}	p_{holm}
Aconchi - Alamos	−1.003	117.837	134.696	0.316	1.000	1.000
Aconchi - Banamichi	−0.719	117.837	129.143	0.472	1.000	1.000
Aconchi - Baviacora	0.084	117.837	116.565	0.933	1.000	1.000
Aconchi - Cananea	0.253	117.837	113.897	0.800	1.000	1.000
Aconchi - Huepac	−0.057	117.837	118.889	0.954	1.000	1.000
Aconchi - San Felipe	2.144	117.837	81.804	0.032*	0.896	0.800
Aconchi - Ures	−0.630	117.837	127.435	0.528	1.000	1.000
Alamos - Banamichi	0.294	134.696	129.143	0.769	1.000	1.000
Alamos - Baviacora	0.982	134.696	116.565	0.326	1.000	1.000
Alamos - Cananea	1.110	134.696	113.897	0.267	1.000	1.000
Alamos - Huepac	0.749	134.696	118.889	0.454	1.000	1.000
Alamos - San Felipe	2.673	134.696	81.804	0.008**	0.211	0.211
Alamos - Ures	0.393	134.696	127.435	0.694	1.000	1.000
Banamichi - Baviacora	0.719	129.143	116.565	0.472	1.000	1.000
Banamichi - Cananea	0.858	129.143	113.897	0.391	1.000	1.000
Banamichi - Huepac	0.506	129.143	118.889	0.613	1.000	1.000
Banamichi - San Felipe	2.507	129.143	81.804	0.012*	0.341	0.329
Banamichi - Ures	0.098	129.143	127.435	0.922	1.000	1.000
Baviacora - Cananea	0.154	116.565	113.897	0.878	1.000	1.000
Baviacora - Huepac	−0.117	116.565	118.889	0.907	1.000	1.000
Baviacora - San Felipe	1.882	116.565	81.804	0.060	1.000	1.000
Baviacora - Ures	−0.638	116.565	127.435	0.524	1.000	1.000
Cananea - Huepac	−0.248	113.897	118.889	0.804	1.000	1.000
Cananea - San Felipe	1.713	113.897	81.804	0.087	1.000	1.000
Cananea - Ures	−0.781	113.897	127.435	0.435	1.000	1.000
Huepac - San Felipe	1.756	118.889	81.804	0.079	1.000	1.000
Huepac - Ures	−0.430	118.889	127.435	0.667	1.000	1.000
San Felipe - Ures	−2.471	81.804	127.435	0.013*	0.377	0.350

* p < .05, ** p < .01

Figure 12

Dunn’s post-hoc comparisons of Behavioral Regulation (BRI) among communities.

Dunn's Post Hoc Comparisons - Community

Comparison	z	W_i	W_j	p	p_{bonf}	p_{holm}
Aconchi - Alamos	−3.189	112.721	167.652	0.001**	0.040*	0.037*
Aconchi - Banamichi	1.131	112.721	94.690	0.258	1.000	1.000
Aconchi - Baviacora	0.835	112.721	99.939	0.404	1.000	1.000
Aconchi - Cananea	−2.213	112.721	148.000	0.027*	0.753	0.538
Aconchi - Huepac	−1.279	112.721	136.778	0.201	1.000	1.000
Aconchi - San Felipe	0.547	112.721	103.304	0.585	1.000	1.000
Aconchi - Ures	−0.114	112.721	114.500	0.909	1.000	1.000
Alamos - Banamichi	3.799	167.652	94.690	< .001***	0.004**	0.004**
Alamos - Baviacora	3.624	167.652	99.939	< .001***	0.008**	0.008**
Alamos - Cananea	1.023	167.652	148.000	0.306	1.000	1.000
Alamos - Huepac	1.426	167.652	136.778	0.154	1.000	1.000
Alamos - San Felipe	3.172	167.652	103.304	0.002**	0.042*	0.038*
Alamos - Ures	2.808	167.652	114.500	0.005**	0.140	0.115
Banamichi - Baviacora	−0.300	94.690	99.939	0.764	1.000	1.000
Banamichi - Cananea	−2.951	94.690	148.000	0.003**	0.089	0.076
Banamichi - Huepac	−2.039	94.690	136.778	0.041*	1.000	0.787
Banamichi - San Felipe	−0.449	94.690	103.304	0.654	1.000	1.000
Banamichi - Ures	−1.115	94.690	114.500	0.265	1.000	1.000
Baviacora - Cananea	−2.745	99.939	148.000	0.006**	0.169	0.133
Baviacora - Huepac	−1.828	99.939	136.778	0.068	1.000	1.000
Baviacora - San Felipe	−0.180	99.939	103.304	0.857	1.000	1.000
Baviacora - Ures	−0.846	99.939	114.500	0.397	1.000	1.000
Cananea - Huepac	0.544	148.000	136.778	0.587	1.000	1.000
Cananea - San Felipe	2.327	148.000	103.304	0.020*	0.559	0.419
Cananea - Ures	1.885	148.000	114.500	0.059	1.000	1.000
Huepac - San Felipe	1.546	136.778	103.304	0.122	1.000	1.000
Huepac - Ures	1.093	136.778	114.500	0.274	1.000	1.000
San Felipe - Ures	−0.591	103.304	114.500	0.554	1.000	1.000

* p < .05, ** p < .01, *** p < .001

Figure 13

Dunn's post-hoc comparisons of Emotional Regulation (ERI) among communities.

Dunn's Post Hoc Comparisons - Community

Comparison	z	W_i	W_j	p	p_{bonf}	p_{holm}
Aconchi - Alamos	−3.737	111.173	175.478	< .001***	0.005**	0.005**
Aconchi - Banamichi	0.487	111.173	103.414	0.626	1.000	1.000
Aconchi - Baviacora	0.994	111.173	95.970	0.320	1.000	1.000
Aconchi - Cananea	−2.943	111.173	158.034	0.003**	0.091	0.068
Aconchi - Huepac	−0.294	111.173	116.694	0.769	1.000	1.000
Aconchi - San Felipe	0.356	111.173	105.043	0.722	1.000	1.000
Aconchi - Ures	0.182	111.173	108.339	0.856	1.000	1.000
Alamos - Banamichi	3.756	175.478	103.414	< .001***	0.005**	0.005**
Alamos - Baviacora	4.260	175.478	95.970	< .001***	< .001***	< .001***
Alamos - Cananea	0.909	175.478	158.034	0.363	1.000	1.000
Alamos - Huepac	2.718	175.478	116.694	0.007**	0.184	0.118
Alamos - San Felipe	3.476	175.478	105.043	< .001***	0.014*	0.012*
Alamos - Ures	3.550	175.478	108.339	< .001***	0.011*	0.010**
Banamichi - Baviacora	0.426	103.414	95.970	0.670	1.000	1.000
Banamichi - Cananea	−3.027	103.414	158.034	0.002**	0.069	0.054
Banamichi - Huepac	−0.644	103.414	116.694	0.520	1.000	1.000
Banamichi - San Felipe	−0.085	103.414	105.043	0.932	1.000	1.000
Banamichi - Ures	−0.277	103.414	108.339	0.781	1.000	1.000
Baviacora - Cananea	−3.549	95.970	158.034	< .001***	0.011*	0.010**
Baviacora - Huepac	−1.029	95.970	116.694	0.303	1.000	1.000
Baviacora - San Felipe	−0.486	95.970	105.043	0.627	1.000	1.000
Baviacora - Ures	−0.720	95.970	108.339	0.472	1.000	1.000
Cananea - Huepac	2.005	158.034	116.694	0.045*	1.000	0.764
Cananea - San Felipe	2.762	158.034	105.043	0.006**	0.161	0.109
Cananea - Ures	2.800	158.034	108.339	0.005**	0.143	0.102
Huepac - San Felipe	0.539	116.694	105.043	0.590	1.000	1.000
Huepac - Ures	0.410	116.694	108.339	0.682	1.000	1.000
San Felipe - Ures	−0.174	105.043	108.339	0.862	1.000	1.000

* p < .05, ** p < .01, *** p < .001

Figure 14

Dunn’s post-hoc comparisons of Cognitive Regulation (CRI) among communities.

Dunn's Post Hoc Comparisons - Community

Comparison	z	W_i	W_j	p	p_{bonf}	p_{holm}
Aconchi - Alamos	−3.656	110.010	173.022	< .001***	0.007**	0.007**
Aconchi - Banamichi	1.067	110.010	92.983	0.286	1.000	1.000
Aconchi - Baviacora	0.519	110.010	102.061	0.604	1.000	1.000
Aconchi - Cananea	−2.792	110.010	154.552	0.005**	0.147	0.110
Aconchi - Huepac	−0.979	110.010	128.444	0.327	1.000	1.000
Aconchi - San Felipe	0.349	110.010	104.000	0.727	1.000	1.000
Aconchi - Ures	−0.166	110.010	112.597	0.868	1.000	1.000
Alamos - Banamichi	4.164	173.022	92.983	< .001***	< .001***	< .001***
Alamos - Baviacora	3.795	173.022	102.061	< .001***	0.004**	0.004**
Alamos - Cananea	0.961	173.022	154.552	0.337	1.000	1.000
Alamos - Huepac	2.058	173.022	128.444	0.040*	1.000	0.713
Alamos - San Felipe	3.400	173.022	104.000	< .001***	0.019*	0.016*
Alamos - Ures	3.190	173.022	112.597	0.001**	0.040*	0.033*
Banamichi - Baviacora	−0.518	92.983	102.061	0.604	1.000	1.000
Banamichi - Cananea	−3.406	92.983	154.552	< .001***	0.018*	0.016*
Banamichi - Huepac	−1.717	92.983	128.444	0.086	1.000	1.000
Banamichi - San Felipe	−0.573	92.983	104.000	0.566	1.000	1.000
Banamichi - Ures	−1.103	92.983	112.597	0.270	1.000	1.000
Baviacora - Cananea	−2.996	102.061	154.552	0.003**	0.077	0.060
Baviacora - Huepac	−1.308	102.061	128.444	0.191	1.000	1.000
Baviacora - San Felipe	−0.104	102.061	104.000	0.917	1.000	1.000
Baviacora - Ures	−0.612	102.061	112.597	0.541	1.000	1.000
Cananea - Huepac	1.264	154.552	128.444	0.206	1.000	1.000
Cananea - San Felipe	2.630	154.552	104.000	0.009**	0.239	0.171
Cananea - Ures	2.359	154.552	112.597	0.018*	0.513	0.348
Huepac - San Felipe	1.128	128.444	104.000	0.259	1.000	1.000
Huepac - Ures	0.777	128.444	112.597	0.437	1.000	1.000
San Felipe - Ures	−0.454	104.000	112.597	0.650	1.000	1.000

* p < .05, ** p < .01, *** p < .001

Figure 15

Dunn's post-hoc comparisons of Attention Correct Responses among communities.

Dunn's Post Hoc Comparisons - Community ▼

Comparison	z	W_i	W_j	p	p_{bonf}	p_{holm}
Aconchi - Alamos	−2.226	113.635	151.795	0.026*	0.728	0.676
Aconchi - Banamichi	0.166	113.635	110.942	0.868	1.000	1.000
Aconchi - Baviacora	0.058	113.635	112.758	0.953	1.000	1.000
Aconchi - Cananea	1.872	113.635	84.054	0.061	1.000	1.000
Aconchi - Huepac	−0.470	113.635	122.306	0.638	1.000	1.000
Aconchi - San Felipe	−0.014	113.635	113.870	0.989	1.000	1.000
Aconchi - Ures	−1.498	113.635	136.548	0.134	1.000	1.000
Alamos - Banamichi	2.092	151.795	110.942	0.036*	1.000	0.884
Alamos - Baviacora	2.104	151.795	112.758	0.035*	0.990	0.884
Alamos - Cananea	3.528	151.795	84.054	< .001***	0.012*	0.012*
Alamos - Huepac	1.377	151.795	122.306	0.169	1.000	1.000
Alamos - San Felipe	1.887	151.795	113.870	0.059	1.000	1.000
Alamos - Ures	0.811	151.795	136.548	0.417	1.000	1.000
Banamichi - Baviacora	−0.103	110.942	112.758	0.918	1.000	1.000
Banamichi - Cananea	1.465	110.942	84.054	0.143	1.000	1.000
Banamichi - Huepac	−0.550	110.942	122.306	0.582	1.000	1.000
Banamichi - San Felipe	−0.152	110.942	113.870	0.879	1.000	1.000
Banamichi - Ures	−1.429	110.942	136.548	0.153	1.000	1.000
Baviacora - Cananea	1.657	112.758	84.054	0.097	1.000	1.000
Baviacora - Huepac	−0.483	112.758	122.306	0.629	1.000	1.000
Baviacora - San Felipe	−0.061	112.758	113.870	0.952	1.000	1.000
Baviacora - Ures	−1.411	112.758	136.548	0.158	1.000	1.000
Cananea - Huepac	−1.879	84.054	122.306	0.060	1.000	1.000
Cananea - San Felipe	−1.572	84.054	113.870	0.116	1.000	1.000
Cananea - Ures	−2.987	84.054	136.548	0.003**	0.079	0.076
Huepac - San Felipe	0.398	122.306	113.870	0.691	1.000	1.000
Huepac - Ures	−0.713	122.306	136.548	0.476	1.000	1.000
San Felipe - Ures	−1.223	113.870	136.548	0.221	1.000	1.000

* p < .05, ** p < .01, *** p < .001

Figure 16

Dunn's post-hoc comparisons of Omission Errors (Attention) among communities.

Dunn's Post Hoc Comparisons - Community

Comparison	z	W_i	W_j	p	p_{bonf}	p_{holm}
Aconchi - Alamos	6.274	142.010	34.455	< .001***	< .001***	< .001***
Aconchi - Banamichi	0.937	142.010	126.846	0.349	1.000	1.000
Aconchi - Baviacora	0.134	142.010	140.000	0.893	1.000	1.000
Aconchi - Cananea	3.282	142.010	90.161	0.001**	0.029*	0.023*
Aconchi - Huepac	1.893	142.010	107.111	0.058	1.000	0.758
Aconchi - San Felipe	−0.597	142.010	152.087	0.550	1.000	1.000
Aconchi - Ures	2.430	142.010	104.839	0.015*	0.422	0.256
Alamos - Banamichi	−4.732	34.455	126.846	< .001***	< .001***	< .001***
Alamos - Baviacora	−5.689	34.455	140.000	< .001***	< .001***	< .001***
Alamos - Cananea	−2.901	34.455	90.161	0.004**	0.104	0.074
Alamos - Huepac	−3.392	34.455	107.111	< .001***	0.019*	0.016*
Alamos - San Felipe	−5.852	34.455	152.087	< .001***	< .001***	< .001***
Alamos - Ures	−3.746	34.455	104.839	< .001***	0.005**	0.004**
Banamichi - Baviacora	−0.744	126.846	140.000	0.457	1.000	1.000
Banamichi - Cananea	1.999	126.846	90.161	0.046*	1.000	0.639
Banamichi - Huepac	0.955	126.846	107.111	0.340	1.000	1.000
Banamichi - San Felipe	−1.308	126.846	152.087	0.191	1.000	1.000
Banamichi - Ures	1.228	126.846	104.839	0.220	1.000	1.000
Baviacora - Cananea	2.878	140.000	90.161	0.004**	0.112	0.076
Baviacora - Huepac	1.665	140.000	107.111	0.096	1.000	1.000
Baviacora - San Felipe	−0.660	140.000	152.087	0.509	1.000	1.000
Baviacora - Ures	2.086	140.000	104.839	0.037*	1.000	0.555
Cananea - Huepac	−0.832	90.161	107.111	0.405	1.000	1.000
Cananea - San Felipe	−3.265	90.161	152.087	0.001**	0.031*	0.023*
Cananea - Ures	−0.835	90.161	104.839	0.404	1.000	1.000
Huepac - San Felipe	−2.120	107.111	152.087	0.034*	0.951	0.543
Huepac - Ures	0.114	107.111	104.839	0.909	1.000	1.000
San Felipe - Ures	2.547	152.087	104.839	0.011*	0.304	0.195

* p < .05, ** p < .01, *** p < .001

Figure 17

Dunn’s post-hoc comparisons of Commission Errors among communities.

Dunn's Post Hoc Comparisons - Community

Comparison	z	W_i	W_j	p	p_{bonf}	p_{holm}
Aconchi - Alamos	−2.758	113.442	160.477	0.006**	0.163	0.105
Aconchi - Banamichi	1.415	113.442	90.654	0.157	1.000	1.000
Aconchi - Baviacora	1.023	113.442	98.182	0.307	1.000	1.000
Aconchi - Cananea	−4.560	113.442	185.981	< .001***	< .001***	< .001***
Aconchi - Huepac	−0.309	113.442	119.111	0.757	1.000	1.000
Aconchi - San Felipe	1.338	113.442	90.978	0.181	1.000	1.000
Aconchi - Ures	1.639	113.442	88.500	0.101	1.000	1.000
Alamos - Banamichi	3.595	160.477	90.654	< .001***	0.009**	0.007**
Alamos - Baviacora	3.375	160.477	98.182	< .001***	0.021*	0.015*
Alamos - Cananea	−1.324	160.477	185.981	0.185	1.000	1.000
Alamos - Huepac	1.941	160.477	119.111	0.052	1.000	0.888
Alamos - San Felipe	3.475	160.477	90.978	< .001***	0.014*	0.011*
Alamos - Ures	3.850	160.477	88.500	< .001***	0.003**	0.003**
Banamichi - Baviacora	−0.428	90.654	98.182	0.669	1.000	1.000
Banamichi - Cananea	−5.174	90.654	185.981	< .001***	< .001***	< .001***
Banamichi - Huepac	−1.384	90.654	119.111	0.166	1.000	1.000
Banamichi - San Felipe	−0.017	90.654	90.978	0.987	1.000	1.000
Banamichi - Ures	0.121	90.654	88.500	0.904	1.000	1.000
Baviacora - Cananea	−5.046	98.182	185.981	< .001***	< .001***	< .001***
Baviacora - Huepac	−1.065	98.182	119.111	0.287	1.000	1.000
Baviacora - San Felipe	0.395	98.182	90.978	0.692	1.000	1.000
Baviacora - Ures	0.577	98.182	88.500	0.564	1.000	1.000
Cananea - Huepac	3.277	185.981	119.111	0.001**	0.029*	0.020*
Cananea - San Felipe	4.993	185.981	90.978	< .001***	< .001***	< .001***
Cananea - Ures	5.522	185.981	88.500	< .001***	< .001***	< .001***
Huepac - San Felipe	1.333	119.111	90.978	0.182	1.000	1.000
Huepac - Ures	1.540	119.111	88.500	0.123	1.000	1.000
San Felipe - Ures	0.134	90.978	88.500	0.893	1.000	1.000

* p < .05, ** p < .01, *** p < .001

Figure 18

Dunn's post-hoc comparisons of Oral Comprehension among communities.

Dunn's Post Hoc Comparisons - Community ▼

Comparison	z	W_i	W_j	p	p_{bonf}	p_{holm}
Aconchi - Alamos	2.108	104.606	69.975	0.035*	0.981	0.841
Aconchi - Banamichi	0.698	104.606	94.000	0.485	1.000	1.000
Aconchi - Baviacora	−1.187	104.606	121.419	0.235	1.000	1.000
Aconchi - Cananea	−0.278	104.606	109.023	0.781	1.000	1.000
Aconchi - Huepac	−1.550	104.606	131.647	0.121	1.000	1.000
Aconchi - San Felipe	−1.002	104.606	120.523	0.316	1.000	1.000
Aconchi - Ures	−0.927	104.606	118.333	0.354	1.000	1.000
Alamos - Banamichi	−1.283	69.975	94.000	0.200	1.000	1.000
Alamos - Baviacora	−2.873	69.975	121.419	0.004**	0.114	0.110
Alamos - Cananea	−2.024	69.975	109.023	0.043*	1.000	0.988
Alamos - Huepac	−2.994	69.975	131.647	0.003**	0.077	0.077
Alamos - San Felipe	−2.620	69.975	120.523	0.009**	0.246	0.225
Alamos - Ures	−2.625	69.975	118.333	0.009**	0.243	0.225
Banamichi - Baviacora	−1.634	94.000	121.419	0.102	1.000	1.000
Banamichi - Cananea	−0.823	94.000	109.023	0.410	1.000	1.000
Banamichi - Huepac	−1.918	94.000	131.647	0.055	1.000	1.000
Banamichi - San Felipe	−1.453	94.000	120.523	0.146	1.000	1.000
Banamichi - Ures	−1.404	94.000	118.333	0.160	1.000	1.000
Baviacora - Cananea	0.712	121.419	109.023	0.476	1.000	1.000
Baviacora - Huepac	−0.543	121.419	131.647	0.587	1.000	1.000
Baviacora - San Felipe	0.052	121.419	120.523	0.959	1.000	1.000
Baviacora - Ures	0.188	121.419	118.333	0.851	1.000	1.000
Cananea - Huepac	−1.122	109.023	131.647	0.262	1.000	1.000
Cananea - San Felipe	−0.611	109.023	120.523	0.541	1.000	1.000
Cananea - Ures	−0.519	109.023	118.333	0.604	1.000	1.000
Huepac - San Felipe	0.552	131.647	120.523	0.581	1.000	1.000
Huepac - Ures	0.689	131.647	118.333	0.491	1.000	1.000
San Felipe - Ures	0.122	120.523	118.333	0.903	1.000	1.000

* p < .05, ** p < .01

Figure 19

Dunn's post-hoc comparisons of Reading among communities.

Dunn's Post Hoc Comparisons - Community

Comparison	z	W_i	W_j	p	p_{bonf}	p_{holm}
Aconchi - Alamos	1.814	96.279	66.458	0.070	1.000	1.000
Aconchi - Banamichi	0.820	96.279	85.609	0.412	1.000	1.000
Aconchi - Baviacora	0.465	96.279	90.313	0.642	1.000	1.000
Aconchi - Cananea	1.228	96.279	77.733	0.219	1.000	1.000
Aconchi - Huepac	0.640	96.279	86.077	0.522	1.000	1.000
Aconchi - San Felipe	0.068	96.279	95.350	0.946	1.000	1.000
Aconchi - Ures	1.142	96.279	81.625	0.253	1.000	1.000
Alamos - Banamichi	−1.068	66.458	85.609	0.285	1.000	1.000
Alamos - Baviacora	−1.340	66.458	90.313	0.180	1.000	1.000
Alamos - Cananea	−0.578	66.458	77.733	0.563	1.000	1.000
Alamos - Huepac	−0.973	66.458	86.077	0.330	1.000	1.000
Alamos - San Felipe	−1.572	66.458	95.350	0.116	1.000	1.000
Alamos - Ures	−0.852	66.458	81.625	0.394	1.000	1.000
Banamichi - Baviacora	−0.320	85.609	90.313	0.749	1.000	1.000
Banamichi - Cananea	0.471	85.609	77.733	0.637	1.000	1.000
Banamichi - Huepac	−0.027	85.609	86.077	0.979	1.000	1.000
Banamichi - San Felipe	−0.633	85.609	95.350	0.527	1.000	1.000
Banamichi - Ures	0.271	85.609	81.625	0.786	1.000	1.000
Baviacora - Cananea	0.759	90.313	77.733	0.448	1.000	1.000
Baviacora - Huepac	0.244	90.313	86.077	0.807	1.000	1.000
Baviacora - San Felipe	−0.330	90.313	95.350	0.741	1.000	1.000
Baviacora - Ures	0.598	90.313	81.625	0.550	1.000	1.000
Cananea - Huepac	−0.437	77.733	86.077	0.662	1.000	1.000
Cananea - San Felipe	−1.024	77.733	95.350	0.306	1.000	1.000
Cananea - Ures	−0.235	77.733	81.625	0.814	1.000	1.000
Huepac - San Felipe	−0.517	86.077	95.350	0.605	1.000	1.000
Huepac - Ures	0.257	86.077	81.625	0.797	1.000	1.000
San Felipe - Ures	0.900	95.350	81.625	0.368	1.000	1.000

Figure 20

Dunn’s post-hoc comparisons of Writing among communities.

Dunn's Post Hoc Comparisons - Community ▼

Comparison	z	W_i	W_j	p	p_{bonf}	p_{holm}
Aconchi - Alamos	−3.010	74.841	126.167	0.003**	0.073	0.068
Aconchi - Banamichi	1.173	74.841	60.262	0.241	1.000	1.000
Aconchi - Baviacora	−0.206	74.841	77.364	0.837	1.000	1.000
Aconchi - Cananea	−1.225	74.841	91.563	0.221	1.000	1.000
Aconchi - Huepac	−0.509	74.841	82.583	0.611	1.000	1.000
Aconchi - San Felipe	−2.051	74.841	101.211	0.040*	1.000	0.845
Aconchi - Ures	0.662	74.841	66.475	0.508	1.000	1.000
Alamos - Banamichi	3.571	126.167	60.262	< .001***	0.010**	0.010**
Alamos - Baviacora	2.663	126.167	77.364	0.008**	0.217	0.186
Alamos - Cananea	1.793	126.167	91.563	0.073	1.000	1.000
Alamos - Huepac	2.134	126.167	82.583	0.033*	0.920	0.723
Alamos - San Felipe	1.332	126.167	101.211	0.183	1.000	1.000
Alamos - Ures	3.211	126.167	66.475	0.001**	0.037*	0.036*
Banamichi - Baviacora	−1.210	60.262	77.364	0.226	1.000	1.000
Banamichi - Cananea	−2.036	60.262	91.563	0.042*	1.000	0.845
Banamichi - Huepac	−1.332	60.262	82.583	0.183	1.000	1.000
Banamichi - San Felipe	−2.792	60.262	101.211	0.005**	0.147	0.131
Banamichi - Ures	−0.429	60.262	66.475	0.668	1.000	1.000
Baviacora - Cananea	−0.933	77.364	91.563	0.351	1.000	1.000
Baviacora - Huepac	−0.314	77.364	82.583	0.753	1.000	1.000
Baviacora - San Felipe	−1.644	77.364	101.211	0.100	1.000	1.000
Baviacora - Ures	0.761	77.364	66.475	0.447	1.000	1.000
Cananea - Huepac	0.508	91.563	82.583	0.612	1.000	1.000
Cananea - San Felipe	−0.614	91.563	101.211	0.539	1.000	1.000
Cananea - Ures	1.615	91.563	66.475	0.106	1.000	1.000
Huepac - San Felipe	−1.091	82.583	101.211	0.275	1.000	1.000
Huepac - Ures	0.952	82.583	66.475	0.341	1.000	1.000
San Felipe - Ures	2.341	101.211	66.475	0.019*	0.539	0.442

* p < .05, ** p < .01, *** p < .001

Figure 21

Dunn’s post-hoc comparisons of Arithmetic Problems among communities.

Dunn's Post Hoc Comparisons - Community ▼

Comparison	z	W_i	W_j	p	p_{bonf}	p_{holm}
Aconchi - Alamos	0.854	80.207	66.833	0.393	1.000	1.000
Aconchi - Banamichi	−0.716	80.207	89.861	0.474	1.000	1.000
Aconchi - Baviacora	−0.499	80.207	86.173	0.618	1.000	1.000
Aconchi - Cananea	1.394	80.207	59.038	0.163	1.000	1.000
Aconchi - Huepac	−0.184	80.207	83.000	0.854	1.000	1.000
Aconchi - San Felipe	−1.018	80.207	93.684	0.309	1.000	1.000
Aconchi - Ures	−0.963	80.207	92.174	0.336	1.000	1.000
Alamos - Banamichi	−1.295	66.833	89.861	0.195	1.000	1.000
Alamos - Baviacora	−1.162	66.833	86.173	0.245	1.000	1.000
Alamos - Cananea	0.408	66.833	59.038	0.683	1.000	1.000
Alamos - Huepac	−0.847	66.833	83.000	0.397	1.000	1.000
Alamos - San Felipe	−1.526	66.833	93.684	0.127	1.000	1.000
Alamos - Ures	−1.492	66.833	92.174	0.136	1.000	1.000
Banamichi - Baviacora	0.252	89.861	86.173	0.801	1.000	1.000
Banamichi - Cananea	1.775	89.861	59.038	0.076	1.000	1.000
Banamichi - Huepac	0.395	89.861	83.000	0.693	1.000	1.000
Banamichi - San Felipe	−0.244	89.861	93.684	0.808	1.000	1.000
Banamichi - Ures	−0.154	89.861	92.174	0.878	1.000	1.000
Baviacora - Cananea	1.674	86.173	59.038	0.094	1.000	1.000
Baviacora - Huepac	0.196	86.173	83.000	0.845	1.000	1.000
Baviacora - San Felipe	−0.522	86.173	93.684	0.602	1.000	1.000
Baviacora - Ures	−0.439	86.173	92.174	0.660	1.000	1.000
Cananea - Huepac	−1.281	59.038	83.000	0.200	1.000	1.000
Cananea - San Felipe	−2.018	59.038	93.684	0.044*	1.000	1.000
Cananea - Ures	−2.002	59.038	92.174	0.045*	1.000	1.000
Huepac - San Felipe	−0.622	83.000	93.684	0.534	1.000	1.000
Huepac - Ures	−0.554	83.000	92.174	0.579	1.000	1.000
San Felipe - Ures	0.102	93.684	92.174	0.919	1.000	1.000

* $p < .05$

Figure 22

Dunn's post-hoc comparisons of Motor Coordination among communities.

Dunn's Post Hoc Comparisons - Community

Comparison	z	W_i	W_j	p	p_{bonf}	p_{holm}
Aconchi - Alamos	1.746	101.459	73.389	0.081	1.000	1.000
Aconchi - Banamichi	−1.217	101.459	118.692	0.223	1.000	1.000
Aconchi - Baviacora	−1.109	101.459	116.621	0.267	1.000	1.000
Aconchi - Cananea	0.955	101.459	84.077	0.340	1.000	1.000
Aconchi - Huepac	1.535	101.459	76.778	0.125	1.000	1.000
Aconchi - San Felipe	−1.229	101.459	119.864	0.219	1.000	1.000
Aconchi - Ures	0.288	101.459	97.426	0.773	1.000	1.000
Alamos - Banamichi	−2.532	73.389	118.692	0.011*	0.317	0.317
Alamos - Baviacora	−2.469	73.389	116.621	0.014*	0.379	0.352
Alamos - Cananea	−0.503	73.389	84.077	0.615	1.000	1.000
Alamos - Huepac	−0.174	73.389	76.778	0.862	1.000	1.000
Alamos - San Felipe	−2.506	73.389	119.864	0.012*	0.342	0.329
Alamos - Ures	−1.354	73.389	97.426	0.176	1.000	1.000
Banamichi - Baviacora	0.131	118.692	116.621	0.895	1.000	1.000
Banamichi - Cananea	1.747	118.692	84.077	0.081	1.000	1.000
Banamichi - Huepac	2.343	118.692	76.778	0.019*	0.536	0.478
Banamichi - San Felipe	−0.069	118.692	119.864	0.945	1.000	1.000
Banamichi - Ures	1.327	118.692	97.426	0.185	1.000	1.000
Baviacora - Cananea	1.671	116.621	84.077	0.095	1.000	1.000
Baviacora - Huepac	2.276	116.621	76.778	0.023*	0.640	0.526
Baviacora - San Felipe	−0.197	116.621	119.864	0.844	1.000	1.000
Baviacora - Ures	1.230	116.621	97.426	0.219	1.000	1.000
Cananea - Huepac	0.344	84.077	76.778	0.731	1.000	1.000
Cananea - San Felipe	−1.753	84.077	119.864	0.080	1.000	1.000
Cananea - Ures	−0.678	84.077	97.426	0.498	1.000	1.000
Huepac - San Felipe	−2.324	76.778	119.864	0.020*	0.564	0.484
Huepac - Ures	−1.163	76.778	97.426	0.245	1.000	1.000
San Felipe - Ures	1.339	119.864	97.426	0.181	1.000	1.000

* $p < .05$

Figure 23

Dunn's post-hoc comparisons of Motor Slowing among communities.

Dunn's Post Hoc Comparisons - Community

Comparison	z	W_i	W_j	p	p_{bonf}	p_{holm}
Aconchi - Alamos	5.613	118.618	33.083	< .001***	< .001***	< .001***
Aconchi - Banamichi	0.966	118.618	104.400	0.334	1.000	1.000
Aconchi - Baviacora	1.828	118.618	94.375	0.068	1.000	1.000
Aconchi - Cananea	0.899	118.618	104.344	0.368	1.000	1.000
Aconchi - Huepac	0.843	118.618	105.778	0.399	1.000	1.000
Aconchi - San Felipe	1.191	118.618	100.789	0.233	1.000	1.000
Aconchi - Ures	4.157	118.618	62.889	< .001***	< .001***	< .001***
Alamos - Banamichi	−4.122	33.083	104.400	< .001***	0.001**	< .001***
Alamos - Baviacora	−3.809	33.083	94.375	< .001***	0.004**	0.003**
Alamos - Cananea	−3.894	33.083	104.344	< .001***	0.003**	0.002**
Alamos - Huepac	−4.095	33.083	105.778	< .001***	0.001**	0.001**
Alamos - San Felipe	−3.865	33.083	100.789	< .001***	0.003**	0.003**
Alamos - Ures	−1.839	33.083	62.889	0.066	1.000	1.000
Banamichi - Baviacora	0.643	104.400	94.375	0.520	1.000	1.000
Banamichi - Cananea	0.003	104.400	104.344	0.997	1.000	1.000
Banamichi - Huepac	−0.080	104.400	105.778	0.937	1.000	1.000
Banamichi - San Felipe	0.212	104.400	100.789	0.832	1.000	1.000
Banamichi - Ures	2.642	104.400	62.889	0.008**	0.231	0.171
Baviacora - Cananea	−0.597	94.375	104.344	0.550	1.000	1.000
Baviacora - Huepac	−0.709	94.375	105.778	0.479	1.000	1.000
Baviacora - San Felipe	−0.405	94.375	100.789	0.685	1.000	1.000
Baviacora - Ures	2.192	94.375	62.889	0.028*	0.795	0.483
Cananea - Huepac	−0.078	104.344	105.778	0.938	1.000	1.000
Cananea - San Felipe	0.197	104.344	100.789	0.844	1.000	1.000
Cananea - Ures	2.467	104.344	62.889	0.014*	0.381	0.259
Huepac - San Felipe	0.285	105.778	100.789	0.776	1.000	1.000
Huepac - Ures	2.646	105.778	62.889	0.008**	0.228	0.171
San Felipe - Ures	2.376	100.789	62.889	0.017*	0.489	0.315

* p < .05, ** p < .01, *** p < .001

Figure 24

Dunn's post-hoc comparisons of Graphesthesia among communities.

Dunn's Post Hoc Comparisons - Community ▼

Comparison	z	W_i	W_j	p	p_{bonf}	p_{holm}
Aconchi - Alamos	−1.469	99.450	123.132	0.142	1.000	1.000
Aconchi - Banamichi	−0.710	99.450	109.712	0.478	1.000	1.000
Aconchi - Baviacora	−0.588	99.450	107.655	0.557	1.000	1.000
Aconchi - Cananea	−0.254	99.450	103.813	0.800	1.000	1.000
Aconchi - Huepac	1.525	99.450	73.250	0.127	1.000	1.000
Aconchi - San Felipe	1.073	99.450	83.283	0.283	1.000	1.000
Aconchi - Ures	−1.792	99.450	124.750	0.073	1.000	1.000
Alamos - Banamichi	0.743	123.132	109.712	0.457	1.000	1.000
Alamos - Baviacora	0.877	123.132	107.655	0.381	1.000	1.000
Alamos - Cananea	0.952	123.132	103.813	0.341	1.000	1.000
Alamos - Huepac	2.458	123.132	73.250	0.014*	0.391	0.371
Alamos - San Felipe	2.149	123.132	83.283	0.032*	0.885	0.790
Alamos - Ures	−0.091	123.132	124.750	0.927	1.000	1.000
Banamichi - Baviacora	0.127	109.712	107.655	0.899	1.000	1.000
Banamichi - Cananea	0.310	109.712	103.813	0.756	1.000	1.000
Banamichi - Huepac	1.919	109.712	73.250	0.055	1.000	1.000
Banamichi - San Felipe	1.544	109.712	83.283	0.123	1.000	1.000
Banamichi - Ures	−0.923	109.712	124.750	0.356	1.000	1.000
Baviacora - Cananea	0.206	107.655	103.813	0.837	1.000	1.000
Baviacora - Huepac	1.847	107.655	73.250	0.065	1.000	1.000
Baviacora - San Felipe	1.460	107.655	83.283	0.144	1.000	1.000
Baviacora - Ures	−1.079	107.655	124.750	0.281	1.000	1.000
Cananea - Huepac	1.445	103.813	73.250	0.148	1.000	1.000
Cananea - San Felipe	1.054	103.813	83.283	0.292	1.000	1.000
Cananea - Ures	−1.117	103.813	124.750	0.264	1.000	1.000
Huepac - San Felipe	−0.515	73.250	83.283	0.606	1.000	1.000
Huepac - Ures	−2.748	73.250	124.750	0.006**	0.168	0.168
San Felipe - Ures	−2.464	83.283	124.750	0.014*	0.385	0.371

* p < .05, ** p < .01

Figure 25

Dunn's post-hoc comparisons of Alternation among communities.

Dunn's Post Hoc Comparisons - Community

Comparison	z	W_i	W_j	p	p_{bonf}	p_{holm}
Aconchi - Alamos	2.722	104.971	61.579	0.006**	0.182	0.156
Aconchi - Banamichi	−0.543	104.971	112.840	0.587	1.000	1.000
Aconchi - Baviacora	−2.052	104.971	132.694	0.040*	1.000	0.763
Aconchi - Cananea	2.019	104.971	69.800	0.044*	1.000	0.784
Aconchi - Huepac	0.614	104.971	94.765	0.539	1.000	1.000
Aconchi - San Felipe	0.854	104.971	91.600	0.393	1.000	1.000
Aconchi - Ures	−0.873	104.971	117.296	0.383	1.000	1.000
Alamos - Banamichi	−2.839	61.579	112.840	0.005**	0.127	0.113
Alamos - Baviacora	−4.115	61.579	132.694	< .001***	0.001**	0.001**
Alamos - Cananea	−0.401	61.579	69.800	0.688	1.000	1.000
Alamos - Huepac	−1.676	61.579	94.765	0.094	1.000	1.000
Alamos - San Felipe	−1.580	61.579	91.600	0.114	1.000	1.000
Alamos - Ures	−3.137	61.579	117.296	0.002**	0.048*	0.044*
Banamichi - Baviacora	−1.245	112.840	132.694	0.213	1.000	1.000
Banamichi - Cananea	2.222	112.840	69.800	0.026*	0.737	0.553
Banamichi - Huepac	0.969	112.840	94.765	0.332	1.000	1.000
Banamichi - San Felipe	1.194	112.840	91.600	0.233	1.000	1.000
Banamichi - Ures	−0.271	112.840	117.296	0.787	1.000	1.000
Baviacora - Cananea	3.371	132.694	69.800	< .001***	0.021*	0.020*
Baviacora - Huepac	2.119	132.694	94.765	0.034*	0.956	0.683
Baviacora - San Felipe	2.415	132.694	91.600	0.016*	0.440	0.346
Baviacora - Ures	0.986	132.694	117.296	0.324	1.000	1.000
Cananea - Huepac	−1.188	69.800	94.765	0.235	1.000	1.000
Cananea - San Felipe	−1.076	69.800	91.600	0.282	1.000	1.000
Cananea - Ures	−2.486	69.800	117.296	0.013*	0.361	0.297
Huepac - San Felipe	0.162	94.765	91.600	0.872	1.000	1.000
Huepac - Ures	−1.227	94.765	117.296	0.220	1.000	1.000
San Felipe - Ures	−1.468	91.600	117.296	0.142	1.000	1.000

* p < .05, ** p < .01, *** p < .001
