# Supplementary figures and images for: Neurodevelopmental impact of mining-related contamination in children from the Sonora river basin
Source: Front Pediatr. 2025 Dec 5;13:1681071. doi: 10.3389/fped.2025.1681071 (PMC12714966; doi:10.3389/fped.2025.1681071)

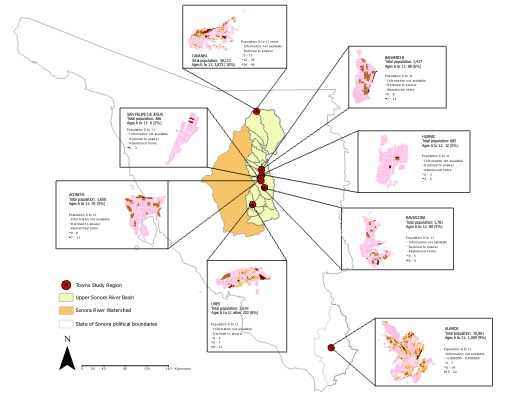

Supplement: Supplementary file 2 [file Image1.jpeg]
